# Messenger RNA in lipid nanoparticles rescues HEK 293 cells from lipid-induced mitochondrial dysfunction as studied by real time pulse chase NMR, RTPC-NMR, spectroscopy

**DOI:** 10.1038/s41598-022-26444-z

**Published:** 2022-12-24

**Authors:** Nicholas Sciolino, Sergey Reverdatto, Aaron Premo, Leonard Breindel, Jianchao Yu, Gregory Theophall, David S. Burz, Anna Liu, Todd Sulchek, Ann Marie Schmidt, Ravichandran Ramasamy, Alexander Shekhtman

**Affiliations:** 1 Department of Chemistry, State University of New York, Albany, NY 12222 USA; 2 Georgia Tech, School of Mechanical Engineering, Atlanta, GA 30332 USA; 3 New York University, Grossman School of Medicine, New York, NY 10016 USA

**Keywords:** Metabolomics, Metabolomics, RNA, NMR spectroscopy, Solution-state NMR

## Abstract

Analytical tools to study cell physiology are critical for optimizing drug-host interactions. Real time pulse chase NMR spectroscopy, RTPC-NMR, was introduced to monitor the kinetics of metabolite production in HEK 293T cells treated with COVID-19 vaccine-like lipid nanoparticles, LNPs, with and without mRNA. Kinetic flux parameters were resolved for the incorporation of isotopic label into metabolites and clearance of labeled metabolites from the cells. Changes in the characteristic times for alanine production implicated mitochondrial dysfunction as a consequence of treating the cells with lipid nanoparticles, LNPs. Mitochondrial dysfunction was largely abated by inclusion of mRNA in the LNPs, the presence of which increased the size and uniformity of the LNPs. The methodology is applicable to all cultured cells.

## Introduction

The remarkable success of pseudouridine-modified mRNA lipid nanoparticle, LNP, -based, vaccines against COVID-19, which harnesses innate translational machinery powered by cellular metabolism, propels this technology to the forefront of medicine^1–6^. Many potential applications of mRNA LNPs to treat chronic diseases such as diabetes^7–9^, cancers and genetic disorders^10,11^ have been tested. Messenger RNA-based therapeutics utilize a complex set of biological molecules that have to be optimized for each application. Can these cells outcompete the surrounding cells for scarce nutrient resources in living tissues? How long will effective production of mRNA gene products last? Will the degradation of mRNA LNPs, which releases methyl pseudouridine, inhibit transcription? Will the lipids that constitute LNPs damage plasma and internal cell membranes? These issues are critical and have to be addressed to facilitate therapeutic implementation.

The dynamic response of cells to drug treatments can be assessed by metabolic profiling, which is generally accomplished by using mass spectroscopy or NMR spectroscopy^12,13^. The methodology typically requires cell lysis and extraction to detect total metabolite concentrations, providing snapshots of cellular physiology over a period of hours to days that is inherently biased due to the multistep processes required to prepare samples^12,13^. The invasive methodology disrupts the compartmental structure of metabolic networks and may not reflect the reactive processes taking place after uptake of the LNP-based vaccine. Real time NMR spectroscopy uses natural ^13^C abundance or tracer-based analyses to identify metabolites by collecting ^13^C-isotope edited ^1^H spectra from cells, allowing for rapid data acquisition, thereby increasing the temporal resolution of the experiments to 10 min or less^14,15^.

More recent real time NMR-based metabolomic studies use a bioreactor to maintain cells in a physiologically active state and can detect free intracellular metabolites, but lack the ability to monitor key reactions such as glycolysis that take place over the course of minutes^16–18^. The introduction of real time pulse chase NMR spectroscopy, RTPC-NMR, combines the use of ^13^C-glucose and ^13^C-edited proton NMR with an ultrasensitive NMR cryoprobe^19^ and the improved design of a bioreactor^20^ to increase the time resolution of the experiments to 47 s allowing rapid changes in metabolic fluxes in response to stimuli to be followed. ^13^C-labeled metabolites are observed against an unlabeled background. This method represents the cutting edge of technology for analyzing cellular health by capturing the dynamics of ^13^C carbon incorporation in an unbiased manner to reveal how stimuli affect metabolic pathways.

## Results

### LNP-transfected mRNA is expressed in HEK 293T cells

**Figure 1:**
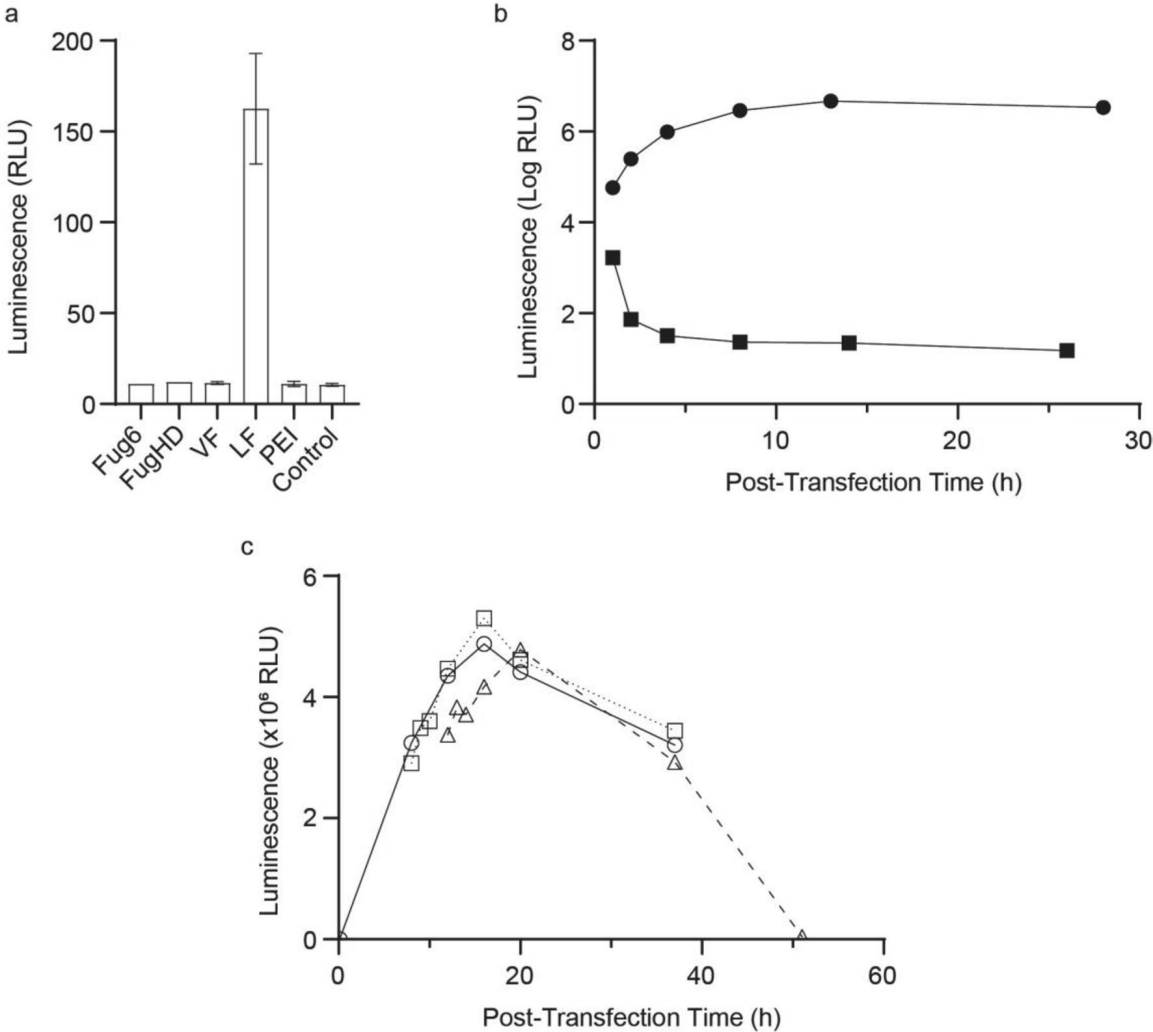
Lipofectamine increases the stability of mRNA transfected into HEK 293T cells. (**a**). Cells were transfected with modified Luciferase mRNA for 24 h using five different reagents and the resulting Luciferase luminescence activity was quantified. The control contained no mRNA and no transfection reagent to assess baseline luminescence. Bars indicate relative error. (**b**). Luminescence resulting from VECT-transfection with naked modified Luciferase mRNA (squares) and LF-mediated transfection with modified Luciferase mRNA (circles). (**c**). Luminescence of HEK 293T cells treated with LF-mRNA for different times. Samples were treated continuously (solid line, circles) or for 8 h (dotted line, squares) or 12 h (dashed line, triangles).

Modified Luciferase mRNA containing N^1^-methylpseudouridine instead of uridine was prepared by in vitro transcription based on a previously published protocol^21,22^ and was used for all experiments. Incorporating N^1^-methylpseudouridine increases mRNA stability and enhances protein expression in mammalian cells^22,23^. The purified transcript included a ~ 150-nucleotide 3’ poly(A) tail and 5’ Cap1^24^ to further facilitate protein translation (Supplementary Figure 1). The modified mRNA was transfected into HEK 293T cells for 24 h using five transfection reagents (Fig. 1a, Supplementary Table 1). HEK 293T cells were previously utilized to monitor the uptake and expression of Luciferase mRNA^21,22^. Consistent with Kariko et al*.*^21,22^, only one reagent, Lipofectamine 2000, LF, delivered sufficient mRNA into the cells to generate a Luciferase-dependent luminescence signal.

The volume exchange for convective transfer, VECT, technique^25,26^ was used to transfect HEK 293T cells with naked mRNA. Despite the chemically modified base, protein production was limited and the Luciferase-dependent signal rapidly diminished (Fig. 1b, Supplementary Table 2). LF-mediated transfection of the same mRNA resulted in sustained protein expression. The data suggest that even modified mRNAs are highly unstable, and that utilizing transfection reagents to protect mRNA from degradation is critical for sustained protein expression.

To establish an analysis window for metabolic studies, the extent of protein expression was examined by incubating HEK 293 T cells with LF-mRNA LNPs for different times. Samples treated continuously or for 8 or 12 h produced comparable amounts of Luciferase-dependent activity over the same time course (Fig. 1c, Supplementary Table 3). Maximum protein expression was observed at ~ 15 h for continuous and 8 h transfections, whereas the 12 h transfection exhibited peak expression at ~ 20 h. All subsequent transfections were incubated for 10 h to maximize protein production and for experimental convenience.

### mRNA cargo increases the average size of lipid nanoparticles

**Figure 2:**
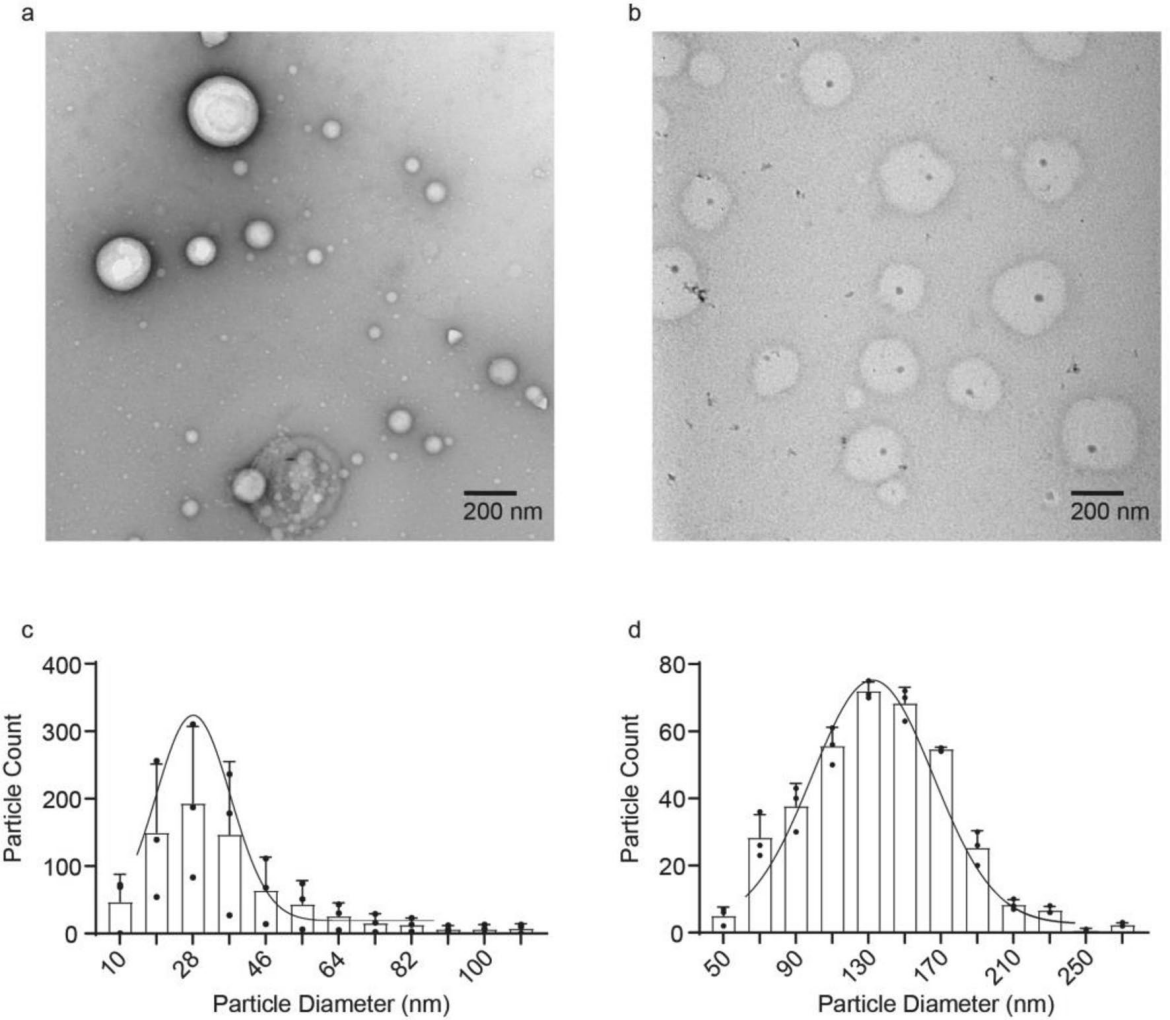
Lipid nanoparticle size is stabilized by mRNA. (**a**) Electron micrograph of liposomes without mRNA. (**b**) Electron micrograph of liposomes containing Luciferase mRNA. (**c**) Size distribution of the LNPs without mRNA. (**d**) Size distribution of the LNPs containing Luciferase mRNA. The Gaussian curve was drawn to ease the comparative visualization of the distribution.

The relative stability and efficacy of LNPs are a function of the particle diameter and dispersity, which control the efficiency of bio-distribution, tissue penetration, cargo loading and release, and cellular uptake in tissues^27–29^. For cultured cells LNP size is expected to play a lesser role in enabling delivery of the cargo due to the absence of circulatory and lymphatic systems which minimize tissue penetration by rapidly removing smaller particles. LNPs form polydisperse liposomal structures with aqueous interiors stabilized by a surfactant bilayer; the aqueous core provides a stable environment for mRNA, reducing premature degradation relative to naked mRNA^30,31^. To ascertain the role of mRNA in the interaction with LF, LNPs were assembled with and without mRNA, and negatively stained with uranyl acetate for electron microscopic imaging (Fig. 2). In the absence of mRNA the LNP size distribution was asymmetric with mean particle size of 29 ± 12 nm (Fig. 2a and c). The inclusion of mRNA cargo in LF LNPs resulted in a larger, less polydisperse population of particle sizes with the mean of 135 ± 12 nm (Fig. 2b and d) that falls within the range, 100–200 nm, of those successfully used to administer cancer drugs to highly permeable tumors^32^.

### Real time pulse-chase NMR spectroscopy

**Figure 3:**
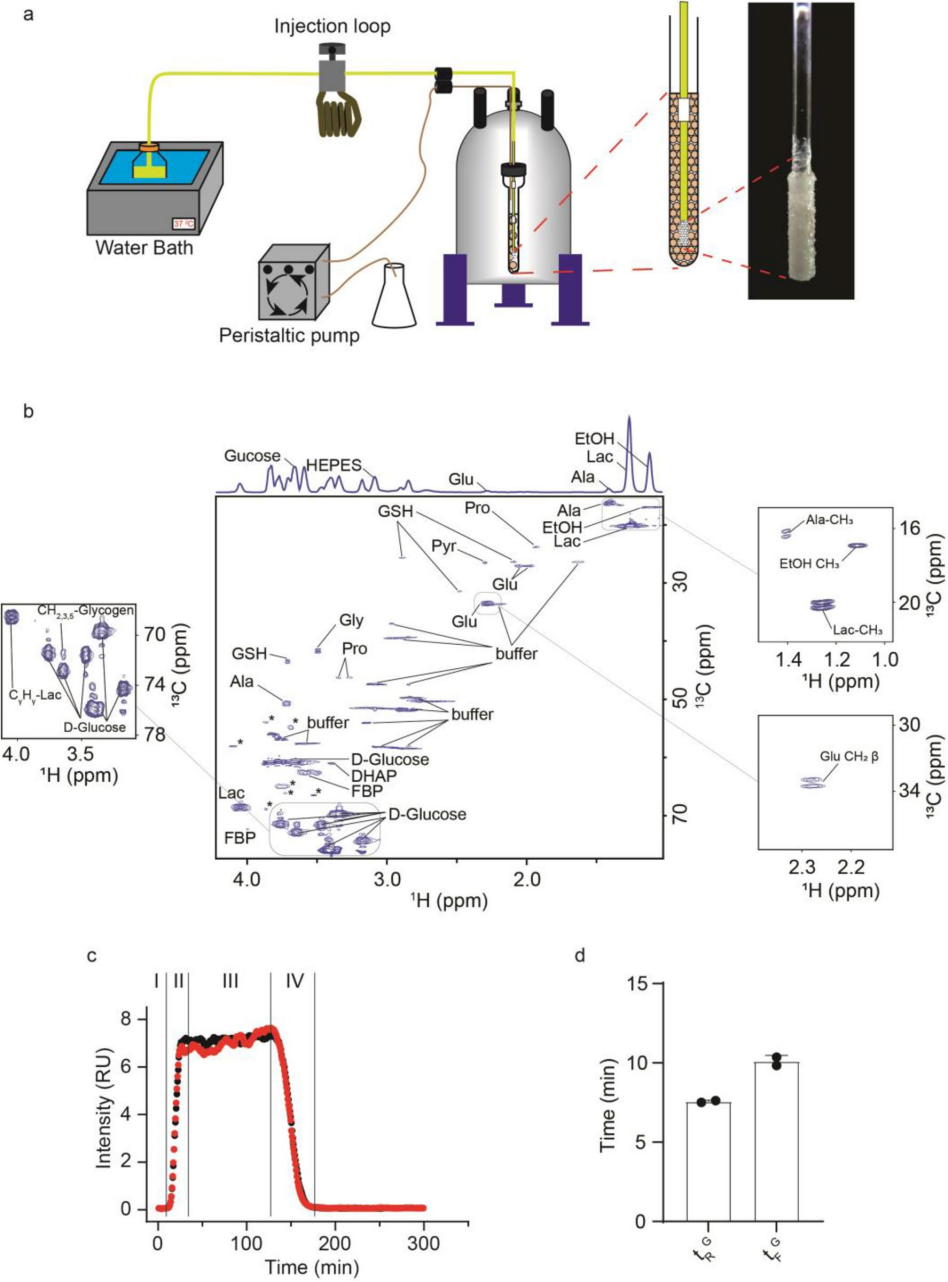
Real time pulse-chase NMR spectroscopy. (**a**) Bioreactor used for RT-PC NMR. The modified bioreactor includes an injectable loop for pulse-chase experiments or a valve for step-chase experiments. (**b**) ^13^C edited heteronuclear single quantum coherence, ^1^H-^13^C HSQC, NMR spectrum showing metabolites present in-cell. Insets show metabolite cross peak splitting and glycogen cross peaks. The projection of the 2D ^1^H-^13^C HSQC into the proton dimension is on the top of the spectrum. The ethanol peak arises because ethanol was used to solubilize the palmitic acid supplement in the modified KH buffer. Unlabeled peaks from metabolites, which were not used for the analysis due to a low signal to noise ratio or overlap with buffer or glucose peaks after projection to the ^1^H axis, are indicated with asterisk. (**c**) Normalized ^13^C-glucose pulses chased with unlabeled glucose in non-transfected HEK 293T cells: (I) Prior to the pulse cells are fed unlabeled glucose in modified KH buffer; (II) During the incorporation stage a 15 mL pulse of ^13^C-glucose is introduced through the injectable loop; (III) the ^13^C-glucose reaches a plateau concentration; (IV) During the clearance stage the ^13^C-glucose is eliminated from the system. During all the stages the individual metabolites are monitored as they progress through metabolic pathways. The red and black traces represent biological replicate samples. (**d**) Rise and fall times for the leading and trailing edges, t_R_^G^ and t_F_^G^, of the pulses shown in panel c.

Pulse-chase capability was added to the previously described real time, RT, NMR bioreactor^33^. The bioreactor maintains high levels of phosphate-containing metabolites for > 24 h, consistent with a high energy charge and metabolically active cells^33^. HEK 293T cells were packaged as previously described into small, ~ 0.6 mm diameter, alginate beads by using an atomizer^20^ and modified Krebs–Henseleit, KH, buffer^34^, a classical chemically defined^35^ serum-free medium used to study cardiomyocytes. KH buffer salts were supplemented with glucose, bovine serum albumin, BSA, insulin, and palmitic acid. The critical feature of the bioreactor is a microporous irrigation stem (Fig. 3a) that permits uniform distribution of the fresh medium across packed cell beads^33^ in 5 mm NMR tube and facilitates quick exchange of the medium from the ~ 200 µL NMR sampling volume. A 15 mL pulse of uniformly labeled [*U*, ^13^C]-glucose was administered through an injection loop attached in line with the medium delivery tubing and controlled by a mechanical switch (Fig. 3a). To avoid the formation of air bubbles, the inner diameter of the tubing was matched with the orifices of the joints throughout the device. Flow was controlled by a peristaltic pump to deliver 100–200 uL/min. The performance of the device was optimized to introduce a pulse of labeled glucose with a rise time, ~ 10 min, that is faster than the characteristic time of glycolysis, 10–20 min^36^. To that end the experimental temperature was adjusted to 300 K to slow cellular metabolism^37^. The optimization runs showed that 10 million cells packaged into alginate beads and fed by using a microporous irrigation stem are able to consume ~ 25% of the ^13^C-glucose administered over the course of the experiment (Supplementary Figure 2).

The ATP level of the cells was monitored before and after each experiment by collecting a phosphorus NMR spectrum. Live cells maintain homeostatic levels of ATP that can be easily discerned in a 1D spectrum. Results showed no reduction in ATP levels over the course of each experiment (Supplementary Figure 3). The intracellular pH was estimated to be between 7.2 and 7.5 based on the difference in the phosphocreatine, PCr, and inorganic phosphate, P_i_, peaks at − 3.18 ppm and 2.13 ppm, respectfully^38,39^. Oxygenation was assessed by the PCr/ATP ratio and was found to be ~ 0.6, which is in the range normally observed for perfused rat kidneys (Supplementary Figure 3)^40^. This ratio did not change over the course of the experiment and showed that in spite of dense packaging in alginate beads the cells were not hypoxic^41,42^.

One dimensional ^13^C-edited proton NMR experiments^43^ were used to monitor carbon that was metabolized from the isotopically labeled glucose^43^. Since proton signals from metabolites overlap, a two dimensional ^13^C-edited heteronuclear single quantum coherence, HSQC^43^, NMR spectrum was first acquired to identify the metabolites present in the proton spectrum (Fig. 3b). Only the peaks from H_γ_–C_γ_ of glutamate, H_β_–C_β_ of alanine and H_γ_–C_γ_ of lactate were not overlapping after projecting to the proton dimension (Fig. 3b). The observed splits in the carbon dimension showed that lactate, alanine and glutamate were products of glycolysis and a single TCA cycle (Fig. 3b insets)^44^. Due to the continuous glucose feed no significant contribution from gluconeogenesis was expected. The non-overlapping peaks corresponding to lactate, alanine, and glutamate were of sufficient strength to reduce the acquisition time to 47 s, which was the temporal resolution of the experiments.

A pulse profile for ^13^C-glucose in untreated HEK 293T cells is shown in Fig. 3c. There was a rapid rise in the signal as the isotopically labeled glucose entered the NMR tube. The signal reached a plateau concentration and decreased as the ^13^C-glucose was chased by unlabeled glucose. The non-ideality of the leading and trailing edges of the glucose pulse are described by the rise and fall times, t_R_^G^ and t_F_^G^, respectively, which were resolved by fitting the pulse-chase profile to a single phase exponential association/decay (Supplementary Figure 4, and Supplementary Table 4). Differential rates of diffusion arise as the pulse enters and leaves the system resulting in significantly longer fall times (Supplementary Figure 5). These differences are in part a result of the variation in cell packing density associated with each physical sample.

### Kinetic flux profiling, KFP, of metabolites

**Figure 4:**
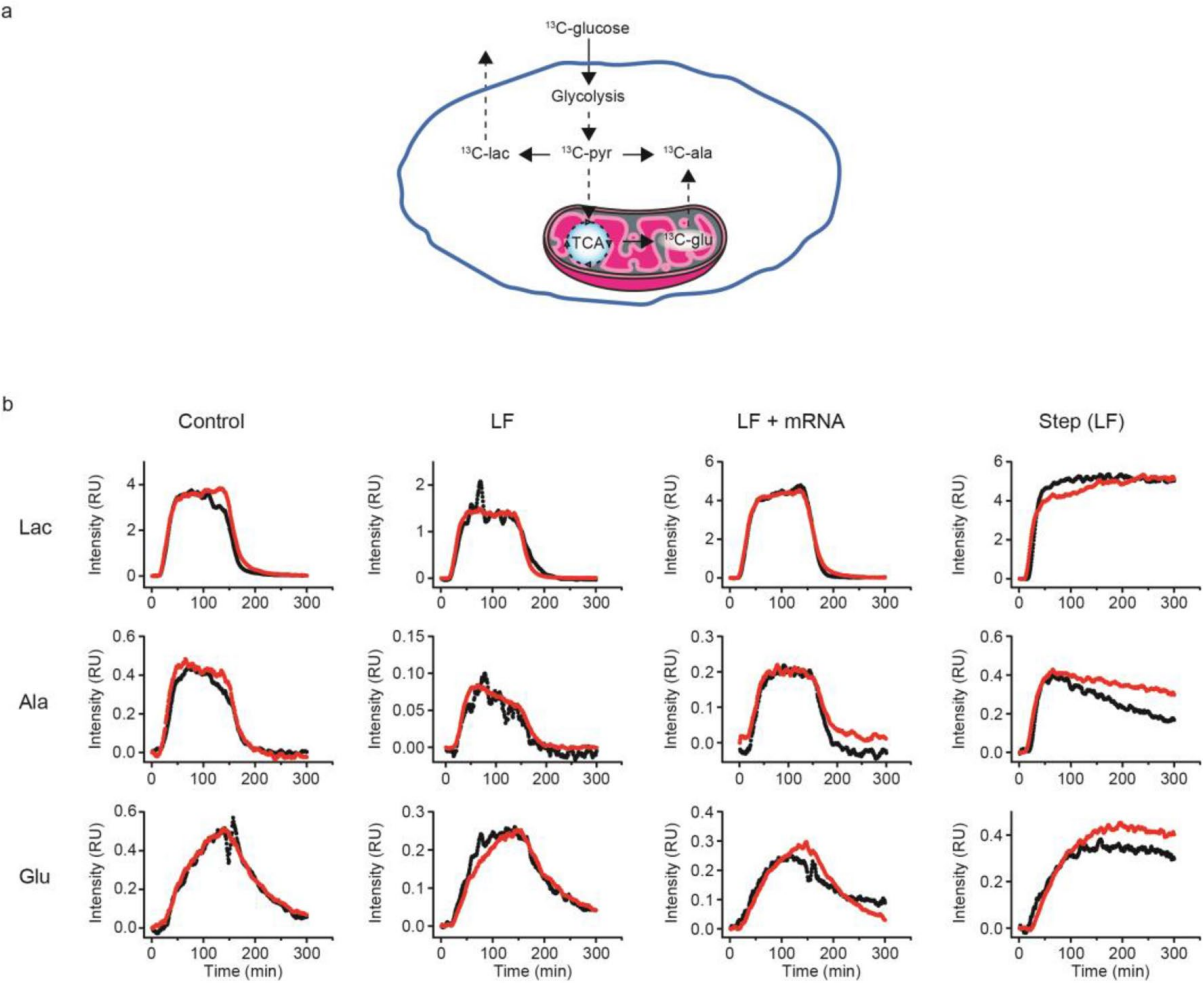
Scaled kinetic flux profiles of metabolites in HEK 293T cells. (**a**) Illustration showing the metabolic disposition of ^13^C-glucose observed during an RTPC-NMR experiment. (**b**) Kinetic flux profiles of metabolites resulting from a 15 mL ^13^C-glucose pulse in HEK 293T cells and a continuous feed of ^13^C-glucose in HEK 293T cells treated for 10 h with LF. The red and black traces represent biological replicate samples.

HEK 293T cells were grown in OptiMEM on plates and either left untreated (control) or treated overnight with LF or LF-mRNA. The fates of lactate, alanine and glutamate were monitored as the labeled glucose was metabolized (Fig. 4). Each experiment took ~ 6 h to complete and was repeated at least twice using biological replicates. Unlike labeled glucose, the maximum cross peak intensities of metabolites varied by a factor of two between individual experiments while the time profile remained unchanged. The variation in the absolute concentration of metabolites with each cell preparation reflects the inherent (clonal) heterogeneity of living cells present even without changes in growth and is not unexpected for biological replicate samples^45,46^. The metabolite cross peak intensities increased as labeled metabolites entered the free pool following biosynthesis and decreased as they were transiently bound or incorporated into larger biomolecules, or cleared from the free pool when labeled glucose was removed from the system.

Under conditions of uniform nutrient flux the leading/trailing edge of the pulse-chase profiles exhibited exponential increases/decreases in the concentration of free metabolite. To estimate kinetic parameters for the production of individual labeled metabolites, KFPs from two experiments were scaled and the leading edges fit to a two-phase exponential that takes into account the real shape of the ^13^C-glucose pulse:

1 $${\text{y }} = {\text{ A}}_{{{\text{Ro}}}} \left( {{1} - {\text{exp}}\left( { - {\text{t}}/{\text{t}}_{{\text{R}}}^{{\text{G}}} } \right)} \right) \, + {\text{ A}}_{{\text{P}}} \left( {{1} - {\text{exp}}\left( { - {\text{t}}/{\text{t}}_{{\text{P}}} } \right)} \right) \, + {\text{ y}}_{{{\text{Po}}}}$$

where y is the relative concentration of free metabolite, t_P_ is the production times required for the metabolite signals to reach ~ 63% saturation, y_Po_ is the relative concentration when the signal begins to increase, and A_Ro_ and A_P_ are constants. To estimate kinetic parameters for the clearance of labeled metabolites from the free pool, the trailing edges of the profiles were also fit to a two-phase exponential:

2 $${\text{y }} = {\text{ A}}_{{{\text{Fo}}}} {\text{exp}}\left( { - {\text{t}}/{\text{t}}_{{\text{F}}}^{{\text{G}}} } \right) \, + {\text{ A}}_{{\text{C}}} {\text{exp}}\left( { - {\text{t}}/{\text{t}}_{{\text{C}}} } \right) \, + {\text{ y}}_{{{\text{Co}}}}$$

where t_C_ is the clearance time for a 63% reduction in the metabolite signals, y_Co_ is the relative concentration when the signal begins to decrease, and A_Fo_ and A_C_ are constants. The first term in Eqs. (1) and (2) takes into account the non-ideality of the ^13^C-glucose pulses due to diffusion (Fig. 3c and d and Supplementary Table 4) when t_R_^G^ and t_F_^G^ are not much shorter than t_P_ and t_C_ of the metabolites.

Control HEK 293T cells that did not undergo LF or LF-mRNA treatment exhibited well-defined leading and trailing edge profiles for lactate, alanine and glutamate (Fig. 4). Lactate levels reached an isotopic steady state^47^ that persisted until the labeled glucose was chased. Alanine exhibited a small steady decline in plateau levels under control conditions. The profile for glutamate reached a maximum about the time the labeled glucose pulse was chased from the system.

The average time constants resolved for production, t_P_, and clearance, t_C_, were 14 and 26 min for lactate and 13 and 30 min for alanine, respectively (Supplementary Table 5, and Supplementary Figures 6 and 7) consistent with the fact that lactate, which is derived from pyruvate, and alanine are products of glycolysis, which is the fastest metabolic pathway and occurs in the cytosol (Fig. 4). Glutamate, which is synthesized in mitochondria from α-ketoglutarate during the TCA cycle exhibited the slowest average times, 75 and 60 min for production and clearance, because of its dependence on the products of glycolysis and the slow import and export of metabolites to and from mitochondria (Supplementary Table 5, and Supplementary Figure 8).

**Figure 5:**
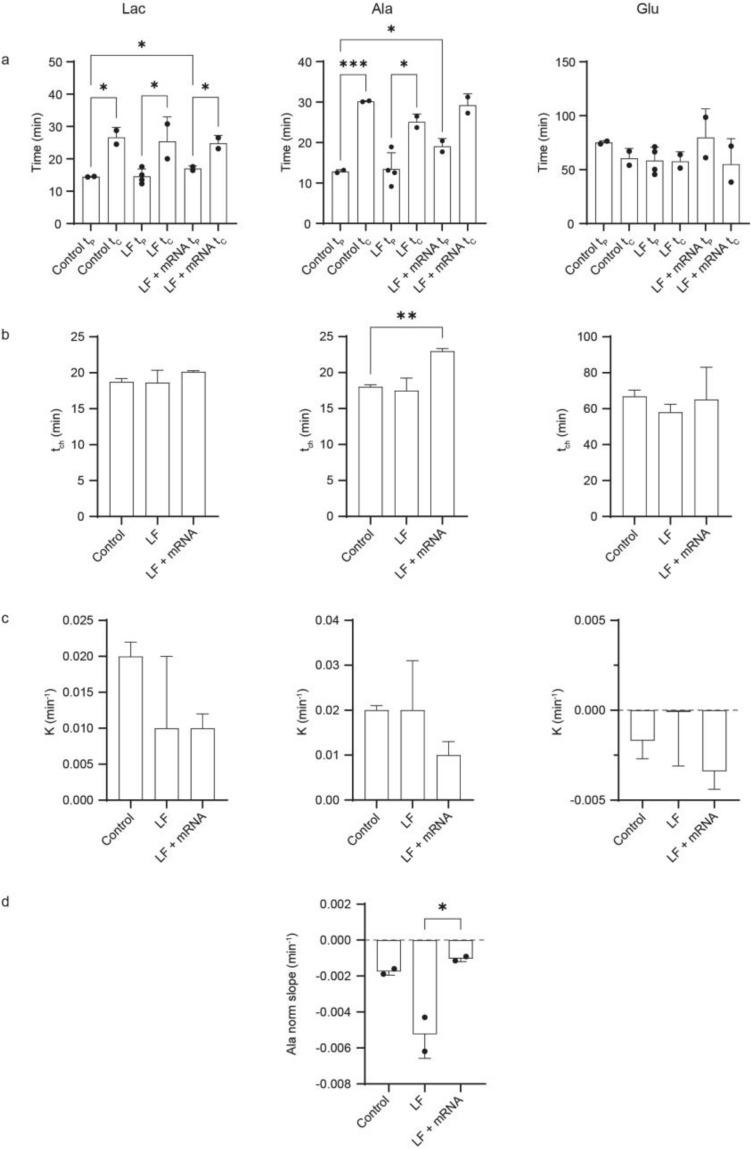
Statistical evaluation of differences between KFP parameters (**a**) production and clearance times, t_P_ and t_C_; (**b**) characteristic times, t_ch_ = 2/(1/t_P_ + 1/t_C_), (**c**) binding parameters, K = (1/t_P_—1/t_C_)/2, for each metabolite, and (**d**) the slopes of alanine KFPs after treatment with LF and LF-mRNA. Dots represent data points obtained using biological replicate samples. Stars indicate significance: *p* < .05 (*), *p* < .01 (**), and *p* < .001 (***).

The kinetic flux profiles of lactate and alanine in cells treated with LF or LF-mRNA were qualitatively similar to controls (Fig. 4**)** exhibiting small but significant increases in t_C_ relative to t_P_ (Supplementary Table 5) due to the bound fraction of labeled metabolite or their precursors (Supplementary Table 5, Fig. 5a). The persistently slower clearance time for lactate and alanine reflected an increase in the concentration of free labeled metabolites as they exchanged out of the bound fraction and were utilized for ongoing metabolism. The differences between t_P_ and t_C_ for glutamate were not significant (Supplementary Table 5, Fig. 5a), most likely because the free intracellular concentration of glutamate is typically ~ 20 times greater than lactate or alanine^48^ such that bound labeled glutamate does not substantially alter the free concentration. The most notable deviation was observed for alanine in cells exposed to LF (Fig. 4) for which the isotopic steady state plateau observed in control cells was replaced by a monotonic decrease in signal strength followed by a rapid exponential decline when the labeled glucose pulse was removed. Isotopic steady state alanine levels were restored when the cells were treated with LF-mRNA.

It should be noted that lactate was also detected in the bioreactor flow through and is therefore excreted from cells (Supplementary Figure 2) but the rate of excretion, ~ 1/20 min^−1^, which is the rate of lactate production (Table 1), is much slower than the nutrient flow rate of 200 uL/min divided by the 200 µL NMR sampling volume (1 min^−1^). As a result, mostly intracellular labeled lactate is observed. The times of lactate and alanine production, t_P_, were significantly slower in LF-mRNA treated HEK 293 T cells as compared to control (Fig. 5a; Supplementary Table 5). Glutamate profiles exhibited consistently higher experimental error in cells treated with LF or LF-mRNA. This could be a result of the sampling noise inherent in biological replicates or an indication of aberrant mitochondrial activity or both.

**Table 1:** Characteristic times (t_ch_) and binding parameters (K) for metabolites.

	Control		LF		LF + mRNA	
	t_ch_ (min)	K (min^−1^)	t_ch_ (min)	K (min^−1^)	t_ch_ (min)	K (min^−1^)
Lac	18.7 ± 0.5	0.020 ± 0.002	18.6 ± 1.7	0.014 ± 0.010	20.1 ± 0.2	0.009 ± 0.002
Ala	18.0 ± 0.3	0.022 ± 0.001	17.5 ± 1.7	0.017 ± 0.011	23.0 ± 0.4	0.009 ± 0.003
Glu	66.8 ± 3.5	− 0.00 ± 0.001	58.0 ± 4.4	− 0.00 ± 0.003	65.1 ± 18.0	− 0.00 ± 0.001

The characteristic time of the metabolic flux, t_ch_, is equal to 2/(1/t_P_ + 1/t_C_). The characteristic binding parameter that describes the rate for incorporation, k_on_[binding sites], of the labeled glucose into and release from the bound fraction, K, is equal to (1/t_P_—1/t_C_)/2. All errors are SEM.

The isotopic steady state incorporation of metabolites into the bound fraction and accumulation of free metabolites under all conditions can be explained by a simple model of competition for binding sites (see Eqs. (14) and (15)). The model predicts the competition between labeled and unlabeled metabolites for binding sites will shorten t_P_ and lengthen t_C_ (Eqs. 11 and 13, Supplementary Table 5, Supplementary Figure 9). In this model the characteristic time of the metabolite flux, t_ch_, is equal to the harmonic mean between t_P_ and t_C_ and the characteristic binding parameter that describes the time for incorporation of the labeled glucose into and release from the bound fraction, K, is equal to half of the difference between 1/t_P_ and 1/t_C_ and is equivalent to the on rate times the concentration of binding sites (Table 1).

No significant differences in t_ch_ were resolved for lactate and glutamate metabolism following treatment with LF or LF-mRNA or for alanine treated with LF (Table 1, Fig. 5) indicating that the glycolytic and mitochondrial metabolic fluxes were unperturbed. There was a significant increase in t_ch_ for alanine metabolism in the presence of LF-mRNA relative to control cells (Fig. 5b). The increase is indicative of a change in alanine metabolism in cells treated with LF-mRNA, which could entail an increased time of utilization of the alanine free pool resulting in a higher bound fraction and/or a decrease in alanine production. However, the K values resolved for each metabolite were not significantly different implying that the metabolic distribution of bound and free metabolites was unaffected by the presence of LF or LF-mRNA (Fig. 5c; Table 1). The negative values resolved for glutamate resulted from the large error associated with those measurements.

To further investigate the perturbations associated with alanine metabolism a step function of labeled glucose was applied to cells treated with LF for 10 h (Fig. 4). The injection loop was replaced with a valve that switched the inlet flow to a reservoir containing ^13^C-glucose medium. Lactate and glutamate displayed isotopic steady state behavior but the profile for alanine reached a maximum value and declined monotonically over the course of the experiment, which lasted approximately twice as long as the pulse experiments. The decrease in plateau signal intensity for alanine was analyzed by fitting the alanine plateau profiles to a linear function (Supplementary Table 6; Fig. 5d). The average value of the slope resolved for cells treated with LF, 0.005 min^−1^, was trending 2.5 times greater than control and, significantly, 5 times greater than in cells treated with LF-mRNA (Supplementary Table 6) indicating a very slow process (Fig. 5d). Because the distribution of bound and free alanine was unperturbed and the small amount of excreted alanine remained constant during the experiment (Supplementary Figure 10), the slope of the alanine plateau most likely reflects a decrease in alanine biosynthesis. The bulk of alanine synthesis relies on the availability of glutamate, which is derived from α-ketoglutarate, a product of the TCA cycle in mitochondria, in the biosynthesis of alanine by amidation of pyruvate and may indicate deficient transport of glutamate out of mitochondria (Fig. 4a). It is also possible that alanine levels may be influenced by rewiring of glutamine metabolism in defective mitochondria^49^, and, if mitochondria are compromised by the generation of reactive oxygen species, ROS, then production of glutamate may also be compromised. Either condition could lead to a reduction in the time of alanine biosynthesis.

### Lipofectamine promotes the formation of ROS in mitochondria

**Figure 6:**
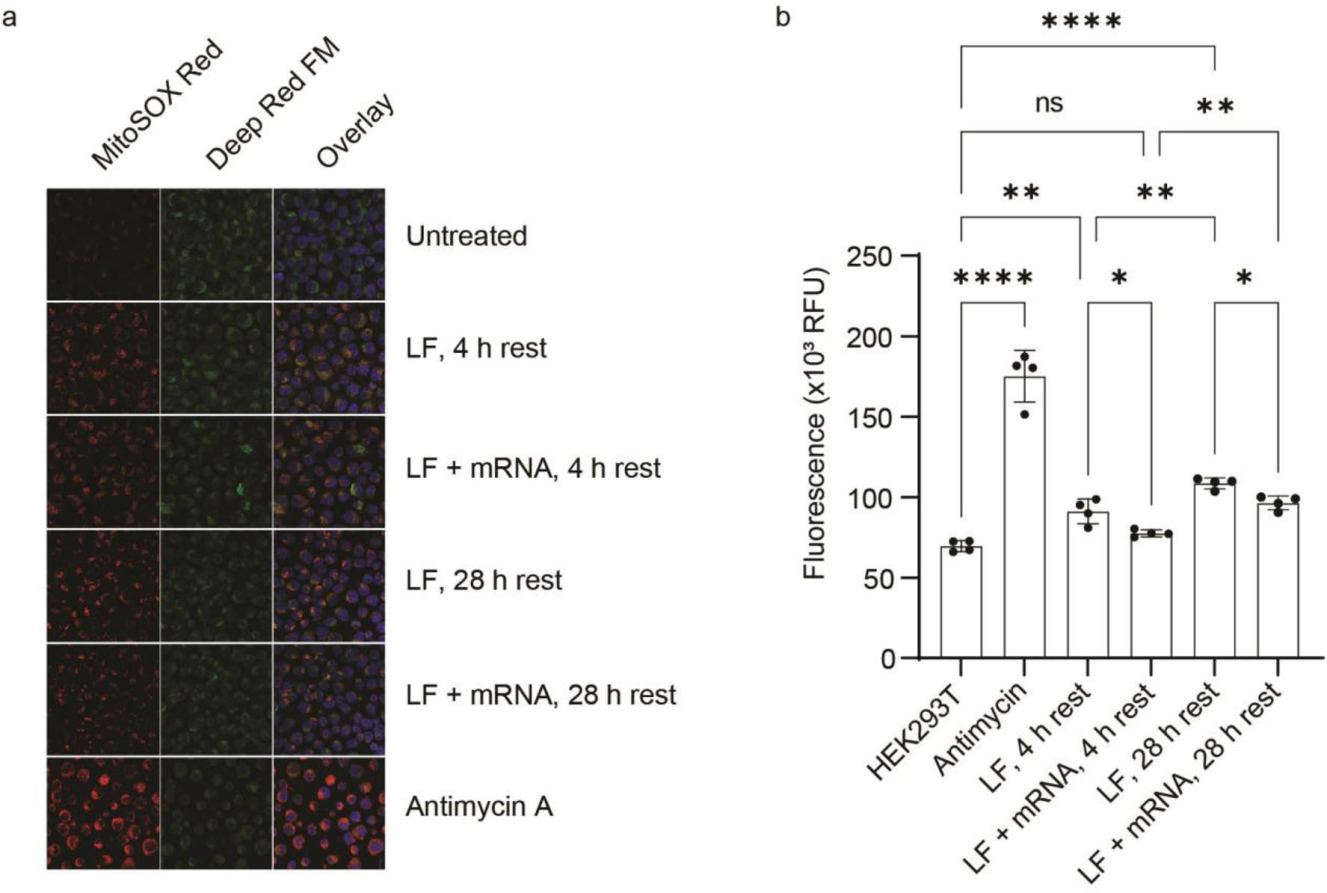
ROS production in HEK 293T cells in response to LNP exposure with and without mRNA. (**a**) MitoSOX stained cells reveal the presence of ROS (colored in red). Deep Red FM staining (colored in green) shows intact mitochondria. Overlay panels indicate that ROS originate in mitochondria. (**b**) Quantitation of MitoSOX fluorescence in cells. Dots represent data points obtained using technical replicate samples. Error bars represent mean deviation. Stars indicate significance: *p* < .05 (*), *p* < .01 (**), and *p* < .001 (***).

Disruption of mitochondrial function, membrane depolarization and electron transport failure, has been observed in neuronal progenitor cells treated with lipofectamine^50^. Electron leakage from the electron transport system on the inner membrane leads to partial reduction of oxygen to form superoxide, an ROS, that can ultimately result in mitophagy. To examine whether mitochondrial function may be affected by LF, HEK 293T cells were treated with MitoSOX, which fluoresces when oxidized by mitochondrial ROS, imaged (Fig. 6a) and assayed (Fig. 6b).

Treating the cells with a positive control for ROS, antimycin A, which binds to the Qi active site on cytochrome c reductase and inhibits cellular respiration, resulted in a significant increase in mitochondrial ROS versus untreated cells (Fig. 6b)^51^. Cells treated with LF and incubated for 4 h exhibited significantly higher levels of ROS compared to untreated cells, and that difference increased with longer, 28 h, incubations. Inclusion of mRNA in the LNP led to significantly lower levels of ROS relative to LF alone, similar to untreated cells after a 4 h incubation (Fig. 6b) and restored the isotopic steady state plateau for alanine in the in-cell RTPC-NMR metabolomics experiments (Fig. 4). Staining for intact mitochondria and dsDNA indicated no differences in mitophagic or autophagic reactions (Fig. 6a). Thus, the apparent decrease in alanine biosynthesis may be due to LF-induced mitochondrial dysfunction, giving rise to ROS.

## Discussion

As LNP-mediated mRNA vaccines become more widely available as a therapeutic treatment, understanding the effect of LNPs on cells and tissues becomes more important^5^. Messenger RNAs alone are too unstable to be used as transfecting agents even those containing modified bases. mRNA needs to be protected by specific LNPs to effectively be utilized by the cell. But LNPs are toxic^50^. The lipid portion of the vaccine analogue appeared to elicit mitochondrial membrane dysfunction in the form of increased ROS, the effects of which were alleviated by the inclusion of mRNA into the LNPs. HEK 293T cells treated with LNPs containing mRNA were able to access nutrients and produce protein for more than 24 h implying that the inevitable release of pseudouridine upon mRNA degradation did not inhibit transcription of the mRNA product. The technique is sensitive to free metabolites but contains information about the covalently-, as in glycogen^52^, or non-covalently-, as in protein–ligand interactions^53^, bound metabolite pool. The use of in-cell metabolomics as an emerging technology to assess the damaging effects of LNPs can help optimize their efficacy and address possible off target effects^54^.

Most metabolic methodologies provide a snapshot of cellular physiology that alters the distribution between free and bound metabolites by disrupting intracellular partitioning and diluting the metabolic pool^36^. Real time pulse chase NMR avoids these complications by introducing a pulse of isotopically labeled ^13^C-glucose into cells within a bioreactor and directly monitoring the times of incorporation and clearance of labeled metabolites inside living cells. By employing one-dimensional ^13^C-edited proton NMR pulse sequences only labeled compounds are observed. NMR signal strength increases when ^13^C-glucose is introduced as intracellular levels of free labeled metabolites are generated and decreases as they are incorporated into intermediary metabolism, transiently bound or cleared from the system when the pulse is removed. The short spectral acquisition time provides a temporal resolution of 47 s, fast enough to observe the onset of rapid processes such as glycolysis.

RTPC-NMR spectroscopy was shown to be effective at monitoring the activity of the energy production pathways in HEK 293T cells under conditions of isotopic steady state nutrient flux in response to an mRNA-LNP vaccine analogue and identifying changes in metabolic fluxes due to the introduction of therapeutic compounds. Three species were well resolved in the proton ^13^C edited NMR spectra, lactate, alanine, and glutamate; these are among the most abundant metabolites inside a cell^48^. The buildup of metabolites followed a well-understood progression of metabolic pathways that participate in extracting cellular energy. The kinetic profiles generated for each was analyzed by using first order kinetics to yield time constants, t_P_ and t_C_, for the production and clearance of labeled metabolites from cells respectively. The experimentally derived parameters resolved characteristic times for metabolic fluxes, t_ch_, as well as the contribution of the bound fraction of metabolites to the flux, K. Lactate and alanine were produced the fastest through glycolysis, which takes place in the cytosol and responds immediately to the presence of glucose. Glutamate synthesis in mitochondria, which relies on the products of glycolysis and the TCA cycle, occurred more slowly (Table 1).

The rapid accumulation of lactate and alanine observed under control conditions were mostly unperturbed by the addition of LNPs with or without mRNA. Consistently slower clearance times resulted from the incorporation of free labeled metabolites or their precursors into the bound fraction, which was slowly released as the pulse was chased from the cells. The significant increase in the characteristic time for alanine in LF-mRNA treated cells is indicative of the previously described metabolic burden of recombinant protein production that results in slower cellular growth^55^ and a decrease in aerobic glycolysis^56,57^. Distinct changes in the plateau profile for alanine clearance implicated mitochondrial dysfunction in glutamate export as a consequence of treating the cells with LNPs alone while glutamate production via the TCA cycle remained unperturbed. This postulate was supported by the presence of ROS in cells treated with LNPs. The extent of ROS was significantly abated, and control behavior was largely restored by including mRNA in the LNPs. The results suggest that alanine may function as a sensitive probe for LNP-induced toxicity.

Incorporation of mRNA into LNPs was shown to be necessary for strong gene expression. Encapsulating mRNA increased the average size of the LNPs from 29 to 135 nm and resulted a more symmetric dispersity, parameters critical for stability and efficacy. In this work the manufacturer’s protocol was used to prepare LNPs with and without mRNA. No attempt was made to vary the size distribution of the resulting particles.

The internalized LNPs diffuse through the cellular medium at a rate that is inversely proportional to their size. We speculate that the rapid diffusion of smaller LNPs coupled with the increased stability afforded by the absence of lipid-RNA interactions^58^ will result in long-lived, deeper penetrating LNPs where they can interact with and damage internal membranous structures including mitochondria. This may explain how empty LNPs elicit greater ROS production than those that are cargo-laden and, in combination with the requirement for proper mitochondrial export of glutamate in the primary biosynthesis of alanine, why only the alanine profiles differed in the absence and presence of mRNA.

## Materials and methods

### Cells and reagents

Human embryonic kidney cells, HEK293T, were purchased from the American Type Culture Collection and cultured according to the recommended conditions. N1-methylpseudouridine-triphosphate was purchased from TriLink Biotechnologies. Transfection reagents Fugene 6, Fugene HD, ViaFect and Luciferase Assay System kit were obtained from Promega. Linear PEI (25 kDa) was purchased from Polysciences, rotenone from MP Biomedicals and Antimycin A from Sigma-Aldrich. Lipofectamine 2000, LF, Dulbecco’s modified Eagle’s medium, DMEM, Opti-MEM reduced serum medium, phosphate buffered saline, PBS, and MitoSOX were from ThermoFisher Scientific. The plasmid pTK305 used for in vitro transcription of firefly Luciferase mRNA was purchased from Addgene (plasmid # 66,812; http://n2t.net/addgene:66812; RRID:Addgene_66812).

### In vitro* RNA *synthesis

Plasmid pTK305 was amplified and linearized with restriction enzyme *Asc*I (New England Biolabs), extracted with phenol/chloroform and precipitated with ethanol/ammonium acetate. Linearized DNA was re-dissolved in RNAse-free Tris-EDTA, TE, buffer at a concentration of 1 mg/mL and served as a template for in vitro transcription using the MEGAscript T7 Transcription Kit (Ambion) with unmodified nucleotides or with N1-methylpseudouridine-triphosphate replacing uridine-triphosphate in the reaction. mRNA synthesis was performed according to the manufacturer’s protocol. The resulting mRNA was purified by precipitation with 7.5 M lithium chloride, 50 mM EDTA, LiCl/EDTA, for ≥ 30 min at − 20 °C. The pellet was dissolved in RNAse-free water.

To introduce the Cap1 structure on the 5’ end, mRNA was denatured at 65 °C for 10 min and the ScriptCap™ Cap1 Capping System (Cellscript) was used according to the manufacturer’s guidelines. The reaction was terminated by adding one half volume of LiCl/EDTA solution and redissolved in RNAse-free water. To add a 3’ poly(A) tail the mRNA was again denatured at 65 °C for 10 min and the A-Plus™ Poly(A) Polymerase Tailing Kit (Cellscript) was used according to manufacturer’s guidelines. The reaction was incubated at 37 °C for 2 h and terminated by adding EDTA to 15 mM. The mRNA was purified by adding an equal volume of 5 M ammonium acetate, incubating on ice and centrifuging the precipitate. The resulting polyadenylated, capped N1-methylpseudouridine mRNA was dissolved in RNAse-free water at concentration of 1 mg/mL, aliquoted and stored at − 70 °C.

### Cell transfections and Luciferase activity assays

HEK 293T cells were grown in complete medium, Dulbecco’s modified Eagle’s medium, DMEM supplemented with 4 mM L-glutamine, 5.5 mM D-glucose, 1 mM sodium pyruvate, 10% fetal bovine serum (HyClone), 100 units/mL of penicillin and 100 mg/mL of streptomycin. To determine transfection efficiency cells were transfected with five different reagents in 24-well plate, 2 wells for each transfection. Each well was seeded with 5 × 10^4^ cells in 0.5 mL of DMEM/10% FBS 24 h before transfection and incubated in a growth chamber in 5% CO_2_ at 37 °C. The growth medium was changed to Opti-MEM/5% FBS and 0.5 µg of luciferase mRNA was combined with either 1.5 µL of FuGENE 6, 1.5 µL of FuGENE HD, 2 µL of ViaFect or 1 µL of LF in Opti-MEM and incubated at room temperature according the manufacturer’s recommendations. 2.5 µL of 1 mg/mL of linear polyethylenimine, PEI, was combined with 0.5 µg of Luciferase mRNA in 47 µL of Opti-MEM and incubated for 20 min at room temperature. Untreated HEK 293T cells were used as an autoluminescent control. Transfection mixtures were transferred to well plates and incubated for 24 h in a growth chamber. Cells were washed with PBS and lysed by adding of 50 µL of 1X Luciferase Cell Culture Lysis Reagent (Promega). Cells were scraped and the well contents were centrifuged at 16,000 g for 5 min. Supernatants were transferred to new tubes and stored at − 70 °C. To assay for luciferase activity 20 µL of lysate was mixed with 100 µL of Luciferase Assay Reagent (Promega) in 96-well white plate (Greiner Bio-One) and immediately read on a Synergy H1 Hybrid Multi-Mode plate reader (BioTek Instruments). Note that LF reagent used in these experiments is composed of a mixture of polycationic and phospholipids. Formation of LNPs containing nucleic acid cargo arises through electrostatic interactions between the phosphate backbone of the nucleic acid polymer and the positively-charged lipids^59^.

For time course experiments, transfected cells were grown on 10 cm plates to 60–80% confluency and harvested with 0.25% trypsin–EDTA (Gibco) for 5–10 min at 37 °C. Trypsin was neutralized by using a fivefold dilution with complete medium, cells were counted and pelleted by centrifugation at 200 g for 10 min. Cells were resuspended in Opti-MEM/5% FBS at a concentration of 0.6–1 × 10^6^ cells/mL and aliquoted into 1.5 mL Eppendorf tubes at 0.5 mL per tube. Tubes were incubated in the growing chamber at an angled position with open lids. For each transfection 2 µL of LF was mixed with 23 µL of Opti-MEM and incubated for 5 min at 37 °C. One µg of mRNA in 25 µL of Opti-MEM was added and incubation continued for 20 min. The transfection mixtures were added to the cells, and tubes were placed in the growth chamber. At the appropriate times cells were centrifuged at 200 g for 5 min, washed with PBS and lysed as described above for the luminescence assay. For some experiments the medium was removed after 8 or 12 h of transfection and replaced with DMEM for further incubation. When microfluidics was used for mRNA delivery (below), the processed cells were resuspended in Opti-MEM/5% FBS, placed into 1.5 mL Eppendorf tubes at 0.5 mL per tube, incubated in the growth chamber for specified times, centrifuged and lysed as above.

Cells for NMR experiments were grown on 15 cm plates in 20 mL of DMEM/10% FBS to 60–80% confluency. Transfection mixtures were scaled up 100-fold, i.e. 200 µL of LF in 2.5 mL of Opti-MEM with or without 100 µg of mRNA. After a 20 min incubation, transfection mixtures were added to the plates, and transfections allowed to proceed in the growth chamber at 37 °C, 5% CO_2_, for 10–11 h.

### Protein transfection by microfluidics

Three 15 cm culture dishes (Corning) containing culture medium were seeded with 4 × 10^6^ HEK 293T cells and incubated in 5% CO_2_ at 37 °C for 48–72 h until ~ 80% confluence (~ 1.2 × 10^7^ cells/plate). Cells were harvested as described above^60^, passed through a 40 µm filter to reduce clumping and aggregation, and counted. ~ 3 × 10^7^ cells were suspended in cell flow buffer, 0.4% BSA, 0.04% EDTA, 20% Percoll and 5 µL of Tween-20 to which modified firefly Luciferase mRNA in storage buffer was added to a final concentration of 300 µM and final volume of 3 mL. A two-channel polydimethylsiloxane, PDMS, Volume Exchange for Convective Transfer, VECT, device with rigid, 9.6 µm microchannels was prepared as previously described^61,62^. The device was purged to remove air and debris using passivation buffer, 1% BSA in PBS, and placed on the stage of a Vista Vision (VWR) microscope for cycle monitoring in the event of bubbles or blockages. The cell suspension was passed through the VECT device using a New Era Pump Systems Model 300 syringe pump at a flow rate of 100 µL/min. The resulting flow through was collected and equilibrated for 20 min at room temperature to maximize transfection and facilitate cell recovery. Cells were centrifuged at 200** g** for 6 min at 25 °C and washed twice with 5 mL PBS for in-cell NMR spectroscopy.

### Transmission electron microscopy

Carbon coated copper microscopy grids (300 mesh, Electron Microscopy Sciences) were glow discharged using air in a Harrick plasma cleaner for 30 s. Liposomes and liposome-mRNA complexes were applied to the freshly discharged grid in 5 µL droplets and left to adsorb for 5 min in a closed 15 cm tissue dish to minimize exposure to air and over-drying. The sample solution was reapplied four times. 2% (w/v) uranyl acetate was prepared and mixed overnight in a dark room on a platform nutator. The grid was stained using 5 µL of 2% uranyl acetate for 1 min deposited directly on the grid and washed 3 times with 5 µL of ultra-pure distilled water. Excess solution was removed by blotting with Whatman paper between each step^63^.

Micrographs were captured using a JEOL JEM-1400 Transmission Electron Microscope, TEM, using a power of 80 kV and an XR401 Charge Coupled Device, CCD, camera (AMT Imaging). Images were captured with a 780 ms exposure time at magnifications of 30,000, 40,000, and 80,000. A total of 17 images containing 1094 particles were processed at low, medium, and high threshold using the program Fiji (ImageJ software). Individual particle areas were measured using the Analyze Particles function. All particles were automatically picked and were manually screened to omit outliers such as particles directly on the border of images or multiple particles that overlapped with each other.

### Cell casting

 ~ 10 × 10^7^ cells (~ 500 µL) were mixed 1:1 (v/v) with Krebs–Henseleit, KH, salts, 50 mM HEPES, pH 7.2, 118 mM NaCl, 4.7 mM KCl, 2.5 mM CaCl_2_, 1.2 mM MgCl_2_, and 3 mM NaH_2_PO_4_ containing 2% alginate (Sigma). The mixture was transferred to a 3 mL syringe and fitted with a Luer-lock tip connected to 40 mm of tygon tubing (I.D. 0.79 mm) with a blunt 21-gauge needle. The needle was oriented at 45° relative to the surface of a 25 mL beaker containing 150 mM CaCl_2_. The cells were injected into an atomizer at 300 µL/min using a syringe pump (New Era Pump System NE-300). The atomizer consisted of a vertically oriented pipette with an airflow of 5.5 L/min. Contact with the CaCl_2_ solution caused the alginate to polymerize into uniformly-sized beads that encapsulated the cells. The CaCl_2_ solution was decanted and replaced with 25 mL of KH salts supplemented with 5 mM glucose, 0.4 mM palmitate, 0.4 mM BSA, and 70 mU/L of insulin. The beads were then transferred to the bioreactor.

### Bioreactor

The bioreactor used to collect metabolic data was almost identical to that described previously^20^ as shown in Fig. 3a. The injector (Rheodyne) was connected to the inlet line 120 cm from a warmed reservoir containing fresh KH salts supplemented with 5 mM ^12^C-glucose and fitted with a 15 mL injection loop constructed out of polyethylene tubing (1.19 mm I.D., Clay Adams). The loop contained KH salts supplemented with 5 mM ^13^C-glucose, allowing for uninterrupted transition to labeled medium. Once the beads were cast and transferred to the NMR tube, the cells were equilibrated with ^12^C glucose in KH medium to allow the beads to settle into their final position within the NMR tube, and to establish a metabolic steady-state prior to the introduction of ^13^C-glucose medium. ^31^P spectra were collected prior to the introduction of labeled medium and at the end of the experiment to assess the energy charge of the cells. The injector valve was switched to initiate the pulse of labeled medium. The labeled metabolite was chased by the continuous flow of unlabeled medium. Flow through was collected in 1.8 mL increments to evaluate cell leakage and metabolite export from the cells by acquiring HSQC spectra separately.

### NMR spectroscopy

All NMR spectra were recorded at 300 K using a 600 MHz Avance III NMR spectrometer equipped with a QCI-P cryoprobe (Bruker). One dimensional ^13^C-isotope edited ^1^H spectra were acquired with 32 scans using ^13^C edited heteronuclear single quantum coherence, HSQC, experiment^43^. The spectral widths in the ^1^H and ^13^C dimensions were 16 and 80 ppm respectively and were digitized by 2048 and 1 points in the ^1^H and ^13^C dimensions, respectively. The time of acquisition for each transient spectrum was 47 s. For each experiment a ^13^C-isotope edited ^1^H spectrum was collected prior to the injection of the ^13^C labeled glucose to establish a baseline reading of the metabolites present in the sample. Following injection, 500 spectra were collected consecutively over a 5.5 h period allowing the disposition of the ^13^C-glucose to be monitored as it progressed through different metabolic pathways. A two dimensional ^13^C-^1^H correlation spectrum was acquired with 32 scans using a ^13^C edited HSQC pulse program^43^. The spectral widths in the ^1^H and ^13^C dimensions were 16 and 80 ppm respectively and were digitized by 2048 and 512 points in the ^1^H and ^13^C dimensions, respectively.

Prior to each trial a proton-decoupled ^31^P spectrum was collected with 5 k scans and 1 s recycle delay to allow the metabolic state of the cell to be assessed. The ^31^P spectrum was centered at − 10 ppm, corresponding to 242.935 MHz, and the spectral width in the ^31^P dimension was 30 ppm. The ^31^P peak intensity at − 11.5 ppm contains α-ATP, α-ADP, NAD^+^ and NAD(H), which are all vital metabolites that can be used to assess cell vitality^20^. All spectra were processed with Topspin version 3.2 (Bruker).

### NMR data analysis

For the metabolic studies, ^1^H-^13^C HSQC spectra were processed by using Topspin (v3.2, Bruker) and MatLab using in-house scripts (Supplementary Methods). Peak intensities were calculated as I = (I/HEPES)–(I_ref_/HEPES_ref_), where I/HEPES represents the normalized peak intensity before the background, I_ref_/HEPES_ref_, was subtracted and HEPES is the peak intensity of HEPES at 3.08 ppm. The number of each HSQC experiment in the series was indexed with a corresponding normalized intensity for glutamate, alanine and lactate at 2.28, 1.41 and 1.26 ppm, respectively. This was plotted in GraphPad Prism to create an *x,y* visualization of the ^13^C-supplemented medium pulse/peak for each metabolite over the course of each experiment.

The change in intensity of each metabolite pulse over time was analyzed. Times for the leading and trailing edges of the pulse were determined from exponential regression models that provided a best fit. The one-phase association model from GraphPad Prism was used to fit ^13^C-glucose intensities on the leading edge of the pulse

3 $${\text{Y }} = {\text{ Y}}_{{\text{o}}} + \, \left( {{\text{Plateau }}{-}{\text{ Y}}_{{\text{o}}} } \right)\left( {{1} - {\text{exp}}\left( { - {\text{Kt}}} \right)} \right)$$

and the one-phase decay model was used to fit the trailing edge

4 $${\text{Y }} = \left( {{\text{Y}}_{{\text{o}}} {-}{\text{ Plateau}}} \right){\text{ exp}}\left( { - {\text{Kt}}} \right) \, + {\text{ Plateau}}$$

where ${\mathrm{Y}}_{\mathrm{o}}$ is the initial intensity of a peak at time zero, Plateau is the maximum (for association) and minimum (for decay) intensity of the peak, K = 1/t_R_^G^ or 1/ t_F_^G^ are the reciprocal rise and fall time constants respectively, and *t* is time in seconds. Since the concentration of intracellular glucose, ~ 0.5 mM^64^, is ten times lower than the concentration of glucose in the KH buffer (5 mM) the contribution of intracellular glucose to the overall glucose NMR signal is negligibly small.

For lactate, alanine, and glutamate the two-phase association model from GraphPad Prism was used to fit intensities on the leading edge of the pulse

5 $${\text{Y }} = {\text{ Y}}_{{\text{o}}} + {\text{ x}}\left( {{\text{Plateau }}{-}{\text{ Y}}_{{\text{o}}} } \right)\left( {{1} - {\text{exp}}\left( { - {\text{t}}/{\text{t}}_{{\text{R}}}^{{\text{G}}} } \right)} \right) \, + \, \left( {{1} - {\text{x}}} \right)\left( {{\text{Plateau }}{-}{\text{ Y}}_{{\text{o}}} } \right)\left( {{1} - {\text{exp}}\left( { - {\text{Kt}}} \right)} \right)$$

where K = 1/t_P_, the reciprocal time constant for metabolite production and 0 ≤ x ≤ 1 is a parameter that assigns the percentage of each exponential component to the overall fit. Note that A_Ro_ and A_P_ in Eq. 1 correspond to x(Plateau –Y_o_) and (1-x)(Y_o_ – Plateau), respectively. The two-phase decay model was used to fit the trailing edge

6 $${\text{Y }} = {\text{ x}}\left( {{\text{Y}}_{{\text{o}}} {-}{\text{ Plateau}}} \right){\text{ exp}}\left( { - {\text{t}}/{\text{t}}_{{\text{F}}}^{{\text{G}}} } \right) \, + \, \left( {{1} - {\text{x}}} \right)\left( {{\text{Y}}_{{\text{o}}} {-}{\text{ Plateau}}} \right){\text{ exp}}\left( { - {\text{Kt}}} \right) \, + {\text{ Plateau}}$$

where K = 1/t_C_, the reciprocal time constant for metabolite clearance. Note that A_Fo_ and A_C_ in Eq. 2 correspond to x (Plateau–Y_o_) and (1-x)(Y_o_–Plateau), respectively. Based on fitting results, x values in (5) and (6) are negligibly small (Supplementary Table 7).

### Kinetic flux profiling of free metabolites

At the leading edge of the ^13^C-glucose pulse, changes in the concentration of free ^13^C-labeled metabolite A^*^, with flux, J and isotopic steady state concentration A, over time t, can be described by a standard kinetic flux profiling, KFP, equation^65,66^ by taking into account the non-ideality of the ^13^C glucose pulse and adding a phenomenological term f_1_(A^*^, t) that describes the possibility that the metabolite is covalently or non-covalently bound to a receptor that is too large to be observed by NMR:

7 $${\text{dA}}^{*} /{\text{dt }} = {\text{ J }}\left( {{1} - {\text{exp}}\left( { - {\text{t}}/{\text{t}}_{{\text{R}}}^{{\text{G}}} } \right)} \right) \, {-}{\text{ J A}}^{*} /{\text{A }} + {\text{f}}_{{1}} \left( {{\text{A}}^{*} ,{\text{ t}}} \right)$$

The equation describes the time evolution of intracellular metabolites such as alanine and glutamate. For excreted metabolites such as lactate, the dilution rate, DR, in the ~ 200 µL NMR sampling volume, V, of the bioreactor equals F/V^67^, where F, the flow rate of the bioreactor is ~ 200 μL/min. The time of dilution, 1/DR ~ 1 min, is much shorter than t_P_ and t_C_, the characteristic times of labeled metabolite production and clearance. Therefore, extracellular metabolites are quickly evacuated from the active volume and do not contribute to the observed NMR signal.

Assuming that the labeled metabolite undergoes chemical exchange between free and bound states in the presence of the unlabeled counterpart and that the concentration of bound metabolites are much smaller than either the total concentration of metabolites or the concentration of binding sites, the f_1_(A^*^, t) term becomes^68^ (see also Eqs. (18) and (21)):

8 $${\text{f}}_{{1}} \left( {{\text{A}}^{*} ,{\text{ t}}} \right) \, = {-}{\text{K A}}^{*} {\text{exp}}\left( {{-}\alpha {\text{t}}} \right)$$

where K is the characteristic binding parameter and α is the characteristic binding rate. Importantly, the term K A^*^ exp(–αt) approaches zero when time goes to infinity, reflecting the fact that no net concentration change due to binding will be observed when the system is equilibrated with labeled metabolites. The solution of this first order differential equation can be found by substituting Eq. (8) into Eq. (7)

9 $${\text{dA}}^{*} /{\text{dt }} + {\text{ A}}^{*} ({\text{J}}/{\text{A }} + {\text{K A}}^{*} {\text{exp}}\left( {{-}\alpha {\text{t}}} \right)) \, = {\text{ J}}\left( {{1} - {\text{exp}}\left( { - {\text{t}}/{\text{t}}_{{{\text{c1}}}}^{{\text{G}}} } \right)} \right)$$

and using the integrating factor, IF^69^

10 $${\text{IF }} = {\text{ exp}}\left( {{\text{J t}}/{\text{A }}{-}{\text{K}}/\alpha {\text{ exp}}\left( {{-}\alpha {\text{t}}} \right)} \right)$$

In general the solution of (9) is possible only numerically. However, the expression for IF is simplified when the metabolite binding is non-specific and αt <  < 1. In this case, the solution of Eq. (10) reduces to

11 $${\text{A}}^{*} = {\text{ y}}_{{{\text{o1}}}} + {\text{ A}}_{{\text{o}}}^{{\text{R}}} ({1}{-}{\text{exp}}({-}{\text{t}}/{\text{t}}_{{\text{R}}}^{{\text{G}}} )) \, + {\text{ A}}_{{1}} \left( {{1}{-}{\text{exp}}\left( {{-}\left( {{\text{J}}/{\text{A }} + {\text{ K}}} \right){\text{ t}}} \right)} \right)$$

where y_o1_, A_o_^R^ and A_1_ are constants determined to fit the kinetic flux profiling at the leading edge (Rise) of ^13^C-glucose pulse. Note that t_P_ = 1/(J/A + K) is the characteristic timescale of the rise in the NMR observed metabolite signal for the leading edge of the ^13^C-glucose pulse and numerically corresponds to the time needed to go from the initial condition to 1/e ~ 0.63 of the saturation level. Importantly, t_P_ is shorter than the characteristic time t_ch_ = A/J^65,66^ for the change in the total concentration of labeled metabolite. This result confirms that intracellular metabolite binding modulates KFP in in-cell NMR based metabolomics.

At the trailing edge of the ^13^C-glucose pulse, changes in the concentration of a free ^13^C-labeled metabolite, A^*^, can also be described by the KFP equation by taking into account the non-ideality of the ^13^C-glucose pulse and adding the phenomenological term f_2_(A^*^, t) = K A^*^ exp(–αt)

12 $${\text{dA}}^{*} /{\text{dt }} = {\text{ J exp}}\left( { - {\text{t}}/{\text{t}}_{{\text{F}}}^{{\text{G}}} } \right) \, {-}{\text{ J A}}^{*} /{\text{A }} + {\text{f}}_{{2}} \left( {{\text{A}}^{*} ,{\text{ t}}} \right)$$

Assuming that the same condition, αt <  < 1, holds true, the solution of Eq. (10) reduces to

13 $${\text{A}}^{*} = {\text{ y}}_{{{\text{o2}}}} + {\text{ A}}_{{\text{o}}}^{{\text{F}}} {\text{exp}}\left( { - {\text{t}}/{\text{t}}_{{\text{F}}}^{{\text{G}}} } \right) + {\text{ A}}_{{2}} {\text{exp}}\left( { - \left( {{\text{J}}/{\text{A }} - {\text{K}}} \right){\text{ t}}} \right)$$

where y_o2_, A_o_^F^ and A_2_ are constants determined to fit the kinetic flux profiling at the trailing edge (Fall) of the ^13^C-glucose pulse. Note that the characteristic time of the clearance in the NMR observed metabolite signal, t_C_ = 1/(J/A-K), is larger than t_P_. In this case, the characteristic time and binding parameters, t_ch_ and K, are defined as t_ch_ = 2/(1/t_P_ + 1/t_C_) and K = (1/t_P_−1/t_C_)/2. This result proves that intracellular metabolite binding leads to asymmetric KFP with respect to the rise and fall of the signal for the ^13^C-glucose pulse and that in-cell NMR can quantify the contribution of metabolite binding to metabolic fluxes in live cells.

### Concentration of free and bound metabolites

A model of competitive binding of labeled metabolites with concentration [A^*^] in the presence of unlabeled metabolites with concentration [A] was used to describe the effect of binding on the concentration of free labeled metabolites^68^. This system is described by two coupled rate equations

14 $${\text{d}}\left[ {{\text{A}}^{*} {\text{R}}} \right]/{\text{dt }} = {\text{ k}}_{{1}} \left[ {{\text{A}}^{*} } \right]\left[ {\text{R}} \right] \, - {\text{ k}}_{{2}} \left[ {{\text{A}}^{*} {\text{R}}} \right]$$

and

15 $${\text{d}}\left[ {{\text{AR}}} \right]/{\text{dt }} = {\text{ k}}_{{1}} \left[ {\text{A}} \right]\left[ {\text{R}} \right] \, - {\text{ k}}_{{2}} \left[ {{\text{AR}}} \right]$$

where [A^*^R] and [AR] are the concentrations of bound labeled and unlabeled metabolites, respectively, k_1_ and k_2_ are on and off rate constants, and [R] is the concentration of free binding sites. If the total concentrations of labeled and unlabeled metabolites and binding sites are [A]_tot_, [A^*^]_tot_, and N, respectively, then [A*] = [A^*^]_tot _− [A^*^R], [A] = [A]_tot_ – [AR] and [R] = N − [A^*^R] − [AR]. Importantly, [A^*^]_tot_ + [A]_tot_ = [A]_ss_, where [A]_ss_ is the concentration of a metabolite at isotopic steady state. In general, (14) and (15) are nonlinear differential equations and can only be solved numerically. However, if the concentration of bound metabolites is much smaller than the total metabolite concentration, Eqs. (14) and (15) are linear and can be solved exactly^68^. The solution depends on initial conditions

16 $$\left[ {{\text{A}}^{*} {\text{R}}} \right] \, = {\text{ N k}}_{{1}} {\text{A}}^{*}_{{{\text{tot}}}} /(\left( {{\text{k}}_{{1}} \left[ {\text{A}} \right]_{{{\text{ss}}}} + {\text{ k}}_{{2}} } \right) \, \times \, \left( {{1} - {\text{exp}}\left( {{-}\left( {{\text{k}}_{{1}} \left[ {\text{A}} \right]_{{{\text{ss}}}} + {\text{ k}}_{{2}} } \right){\text{ t}}} \right)} \right)$$

when [A^*^R] _t = 0_ = 0 and

17 $$\left[ {{\text{A}}^{*} {\text{R}}} \right] \, = {\text{ N k}}_{{1}} {\text{A}}^{*}_{{{\text{tot}}}} /\left( {{\text{k}}_{{1}} \left[ {\text{A}} \right]_{{{\text{ss}}}} + {\text{ k}}_{{2}} } \right) \times {\text{exp}}\left( {{-} \, \left( {{\text{k}}_{{1}} \left[ {\text{A}} \right]_{{{\text{ss}}}} + {\text{ k}}_{{2}} } \right){\text{ t}}} \right)$$

when $$\left[ {{\text{A}}^{*} {\text{R}}} \right]_{{{\text{t }} = \, 0}} = {\text{ N k}}_{{1}} {\text{A}}^{*}_{{{\text{tot}}}} /\left( {{\text{k}}_{{1}} \left[ {\text{A}} \right]_{{{\text{ss}}}} + {\text{ k}}_{{2}} } \right)$$.

Importantly, the change in the concentration of bound metabolite over time can be described by differentiating (14) and (15), and presenting the result as

18 $${\text{d}}\left[ {{\text{A}}^{*} {\text{R}}} \right]/{\text{dt }} = \, \pm {\text{K A}}^{*}_{{{\text{tot}}}} {\text{exp}}\left( { - \alpha {\text{t}}} \right)$$

where K = N k_1_ and α = k_1_ [A]_ss_ + k_2_. Negative d[A^*^R]/dt defines f_1_(A^*^, t) in Eq. (7) and corresponds to the initial conditions [A^*^R]_t = 0_ = 0 and positive d[A^*^R]/dt defines f_2_(A^*^, t) in Eq. (12) and corresponds to the initial condition [A^*^R]_t = 0_ = N k_1_ A^*^_tot_/(k_1_ [A]_ss_ + k_2_).

Equations (14) and (15) are also linear when the concentration of bound metabolites are much smaller than the total concentration of binding sites, N. In this case, the solution also depends on initial conditions

19 $$\left[ {{\text{A}}^{*} {\text{R}}} \right] \, = {\text{ N k}}_{{1}} {\text{A}}^{*}_{{{\text{tot}}}} /\left( {{\text{k}}_{{1}} {\text{N }} + {\text{ k}}_{{2}} } \right) \, \times \, \left( {{1 }{-}{\text{exp}}\left( { - \left( {{\text{k}}_{{1}} {\text{N }} + {\text{ k}}_{{2}} } \right){\text{ t}}} \right)} \right)$$

when [A^*^R] _t = 0_ = 0 and

20 $$\left[ {{\text{A}}^{*} {\text{R}}} \right] \, = {\text{ N k}}_{{1}} {\text{A}}^{*}_{{{\text{tot}}}} /\left( {{\text{k}}_{{1}} {\text{N }} + {\text{ k}}_{{2}} } \right) \, \times {\text{ exp}}\left( {{-}\left( {{\text{k}}_{{1}} {\text{N }} + {\text{ k}}_{{2}} } \right){\text{ t}}} \right)$$

when [A^*^R]_t = 0_ = N k_1_ A^*^_tot_/(k_1_ N + k_2_). Importantly, the change in the concentration of bound metabolite over time can also be described by differentiating (19) and (20) and presenting the result as

21 $${\text{d}}\left[ {{\text{A}}^{*} {\text{R}}} \right]/{\text{dt }} = \, \pm {\text{K }} \times {\text{ A}}^{*}_{{{\text{tot}}}} \times {\text{ exp}}\left( { - \alpha {\text{t}}} \right)$$

where K = N k_1_, and α = k_1_ N + k_2_. In this case, negative d[A^*^R]/dt defines f_1_(A^*^, t) and corresponds to the initial conditions [A^*^R]_t = 0_ = 0 and positive d[A^*^R]/dt defines f_2_(A^*^, t) and corresponds to the initial condition [A^*^R]_t = 0_ = N k_1_ A^*^_tot_/(k_1_ N + k_2_). [A^*^R]_t = 0_ = N k_1_ A^*^_tot_/(k_1_ [A]_ss_ + k_2_). Numerical solutions for Eqs. (14) and (15) in the general case when [AR] or [A^*^R] are comparable to [A]_tot_ or [A^*^]_tot_ is presented in Supplementary Figure 5. These equations can always be fit using an exponential function like Eq. (18) with an R^2^ factor better than 0.99 indicating that an exponential is a good approximation even in the general case.

### Assay for ROS

Six-well plates were seeded with 3 × 10^5^ HEK 293T cells in 2 mL of Opti-MEM/5% FBS medium and incubated in 5% CO_2_ for 20 h at 37 °C. Ten µL of LF in 200 µL of OptiMEM was added to the first well and 5 µg of modified Luciferase mRNA plus 10 µL of LF in 200 µL of OptiMEM to a second well. The transfections were incubated in a growth chamber for 5 h in 5% CO_2_ at 37 °C. The medium was replaced with 2 mL of pre-warmed OptiMEM/5% FBS and the cells allowed to recover for 24 h in the growth chamber (28 h rest). The process was repeated for another two wells and the plate incubated for 4 h in the growth chamber (4 h rest). Before the end of the 4 h incubation, Antimycin A, which inhibits the electron transport complex, was added to another well at concentration of 1.5 µM and incubated for 1 h. The well was washed once with warm OptiMEM/5% FBS and replenished with 2 mL of the same medium. Cells were harvested from the wells with trypsin–EDTA as described above and transferred to 1.5 mL Eppendorf tubes, washed twice with warm modified PBS, PBS containing 1 mM MgCl_2_, 1 mM CaCl_2_ and 5.5 mM glucose. Lastly, all wells were stained with 4 μM MitoSOX in modified PBS for 30 min in the growth chamber. Cells were centrifuged at 300** g** for 5 min and washed twice with modified PBS and re-centrifuged. The cells were re-suspended in 80 µL of modified PBS and 10 µL were aliquoted into each of four wells of a black 384w plate (# 784,086, Greiner Bio-One). The plate was read on a Synergy H1 plate reader (BioTek) with excitation at 510 nm and emission at 580 nm. Ten µL of each cell suspension was diluted into 245 µL of PBS and the number of cells counted manually using a hemocytometer.

### Cell imaging

Fifteen mm diameter round glass coverslips (Ted Pella) were coated with Cell-Tak solution (Corning) according to manufacturer’s instructions. HEK 293T cells were grown and treated like the ROS plate assays with the following modifications. After the cells were transfected, medium refreshed and rested for 28 or 4 h, all wells were stained by adding Mito Tracker Deep Red FM to a final concentration of 50 nM and incubated for 30 min at 37 °C. The wells were washed with PBS and the cells harvested with 400 µL of trypsin/EDTA, neutralized with 2 mL of OptiMEM/5%FBS, centrifuged, washed twice with modified PBS, re-suspended in Eppendorf tubes in the same buffer containing 4 µM MitoSOX. The cells were incubated at 37 °C for 10 min, washed and resuspended in 300 µL of modified PBS and added to the Cell-Tak-treated coverslips for attachment in the growth chamber for 20 min. One of the wells, containing non-transfected HEK 293T cells was treated with 1 µM of Antimycin-A. After attachment all wells were washed twice with PBS. Three hundred µL of 4% formaldehyde was added and the cells were fixed for 10 min at 37 °C. The cells were washed with PBS and incubated for 10 min with 1 μg/mL Hoechst 33,258 dye in PBS at room temperature. The coverslips were washed twice with PBS and mounted on glass slides using Fluoromount G (Electron Microscopy Sciences). Cells were imaged the next day using a Zeiss LSM 710 laser scanning confocal microscope (Carl Zeiss Microscopy) with a 63 × objective and analyzed with ImageJ (USA National Institute of Health, https://imagej.nih.gov/ij/).

### Statistical analysis

To establish statistical significance between average resolved parameters unpaired t-test were performed using Prism (GraphPad).

## Supplementary Information

Supplementary Information.

## Data Availability

All data generated or analysed during this study are included in this published article and its supplementary information file.
