# Supplementary material for: Messenger RNA in lipid nanoparticles rescues HEK 293 cells from lipid-induced mitochondrial dysfunction as studied by real time pulse chase NMR, RTPC-NMR, spectroscopy
Source: Sci Rep. 2022 Dec 24;12:22293. doi: 10.1038/s41598-022-26444-z (PMC9789524; doi:10.1038/s41598-022-26444-z)
Supplement: Supplementary file 1 — Supplementary Information. [file 41598_2022_26444_MOESM1_ESM.pdf]

Supplementary Information

Supplementary Methods. NMR data analysis.

Supplementary Figure 1. Construction of modified *Luciferase* mRNA including unedited image of the gel.

Supplementary Figure 2. Bioreactor flow through.

Supplementary Figure 3. Cellular energy levels during RTPC-NMR.

Supplementary Figure 4. Leading and trailing edge analysis of glucose pulse profiles.

Supplementary Figure 5. Statistical analysis of glucose pulses.

Supplementary Figure 6. Leading and trailing edge analysis for lactate profiles.

Supplementary Figure 7. Leading and trailing edge analysis for alanine profiles.

Supplementary Figure 8. Leading and trailing edge analysis for glutamate profiles.

Supplementary Figure 9. Concentrations of bound labeled metabolites as a function of binding site concentration.

Supplementary Figure 10. Alanine excreted from cells treated with LF.

Supplementary Table 1. Raw data for Figure 1a.

Supplementary Table 2. Raw data for Figure 1b.

Supplementary Table 3. Raw data for Figure 1c.

Supplementary Table 4. Characteristic times for the glucose pulses.

Supplementary Table 5. t_P and t_C values resolved from metabolite profiles.

Supplementary Table 6. Slopes of alanine plateau profiles.

Supplementary Table 7. Ratios of A_{Ro}/A_P and A_{Fo}/A_C.

Supplementary Methods

NMR data analysis

The *multicmd* command was used to create a script to automatically process 1D spectra.

```
multicmd
number of experiments
si 2k
stsr 1k
stsi 512
ft 1 999
```

si sets the size of the real spectrum for 1D data; *stsr* sets the first output points of a strip transform, taking an integer value between this and *si*. *stsi* is the strip size, setting the number of output points of the strip transformation. *ft* is Fourier Transform to transform a 1D data set with dimension ≥2.

MatLab was used to automatically relabel and move the resulting data to a new location to be processed in TopSpin.

```
i=1;
j=starting experiment number
while i < 67
   s=int2str(i);
   ss=int2str(j);
   ll=['Combined FT/' ss '/pdata/999'];
   l=['folder path/' s '/pdata/1'];
   movefile(ll, l, 'f')
   i=i+1;
   j=j+1;
end
```

The *multicmd* command was again used to automatically perform phase and baseline corrections on the new spectral set, followed by manual adjustment of each spectrum to refine corrections to all positive values if necessary.

```
multicmd
number of experiments
apk
abs
```

The Bruker Topspin 3.1 *convbin2asc* command was used to create an ascii file with the intensities and ppm range for each experiment. MatLab was used to extract the resulting refined intensities and create an MS Excel file for normalization and data analysis.

```
cd folder address/1/pdata/1
A = dlmread('ascii-spec.txt', ',', 1, 0);
B = [A(:,4), A(:,2)];
i=1;
while i<67
   cd ../../../../
   s=int2str(i);
   l=['folder address/' s '/pdata/1'];
   cd(l)
   A = dlmread('ascii-spec.txt', ',', 1, 0);
   C = [B,A(:,2)];
   B = C;
   i=i+1;
end
cd ../../../../
csvwrite('file name.csv', B)
```

Supplementary Figures

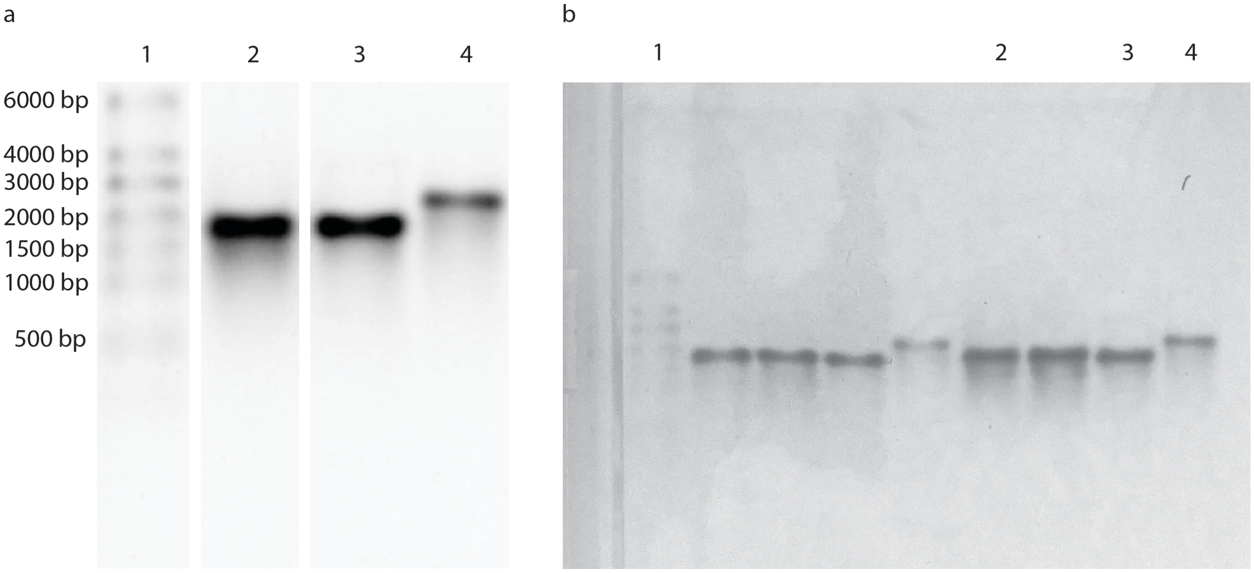

Supplementary Figure 1. Construction of modified *luciferase* mRNA including unedited image of the gel. a) Bleach gel (Aranda *et al.*, 2012 *Electrophoresis* **33**, 366) showing the construction of modified *luciferase*, *luc*, mRNA containing 1-methylpseudouridine, an ~150-nucleotide 3' poly(A) tail and 5' Cap-1. Lane 1 is mRNA ladder. Lane 2 is *luc* mRNA(1650 nt) synthesized with regular ribonucleotide triphosphates. Lane 3 is *luc* mRNA synthesized with N1-methylpseudouridine-triphosphate replacing uridine triphosphate in the reaction. Lane 4 is polyadenylated N1-methylpseudouridine *luc* RNA supplemented with a 5'-Cap 1 structure. b) The unedited image of the gel lanes presented in a). Lanes used to assemble panel a, are indicated.

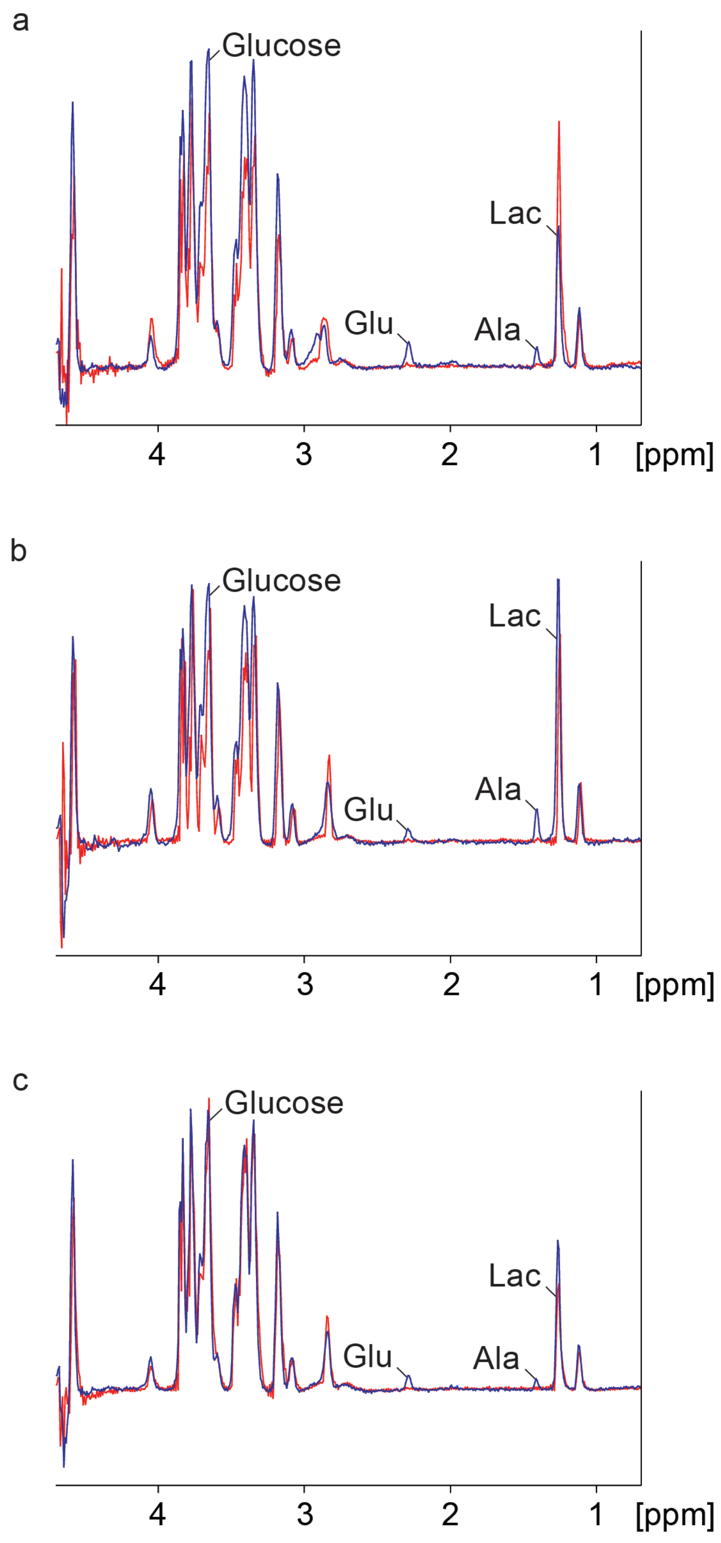

Supplementary Figure 2. Bioreactor flow through. Overlays of in-cell ^{13}C-edited proton spectra (blue) and flow through (red) spectra for a) untreated HEK 293T cells; b) cells treated with lipofectamine, and; c) cells treated with lipofectamine-mRNA. In-cell spectra and 1.8 mL aliquots

of flow through were collected midway through the experiment. The spectra of the flow through samples were acquired at the end of the experiment. Glu is glutamate, Ala is alanine and Lac is lactate. Only lactate was evident in the flow through. The decrease in the area of the glucose cross peak over the course of the experiment provides an estimate of the extent of glucose consumption.

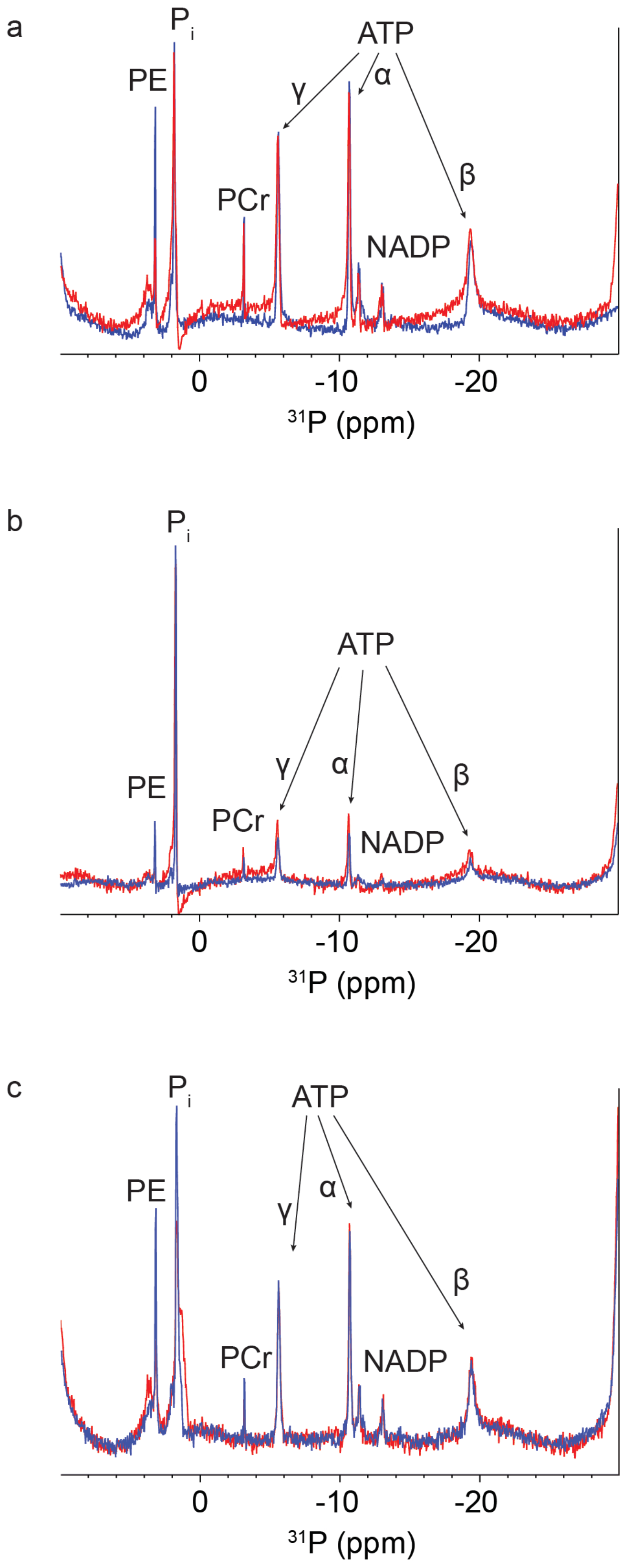
a
P i
PE
ATP
γ
α
PCr
β
NADP
0
-10
-20
31P (ppm)
b
P i
ATP
γ
α
β
PE
PCr
NADP
0
-10
-20
31P (ppm)
c
P i
ATP
PE
γ
α
β
PCr
NADP
0
-10
-20
31P (ppm)

Supplementary Figure 3. Cellular energy levels during RTPC-NMR. Overlays of in-cell ^{31}P spectra after equilibration in modified KH buffer before the ^{13}C-glucose pulse (blue) and after the labeled medium was removed (red). a) untreated HEK 293T cells; b) cells treated with LF; c) cells treated with LF-mRNA. PE is phosphoester, P_i is monophosphate. PCr is phosphocreatine, and α, β, and γ are phosphorus in ATP. The ATP peaks are nearly superimposed indicating that cell viability did not diminish over the course of the experiments. The peaks for intracellular and extracellular P_i overlap.

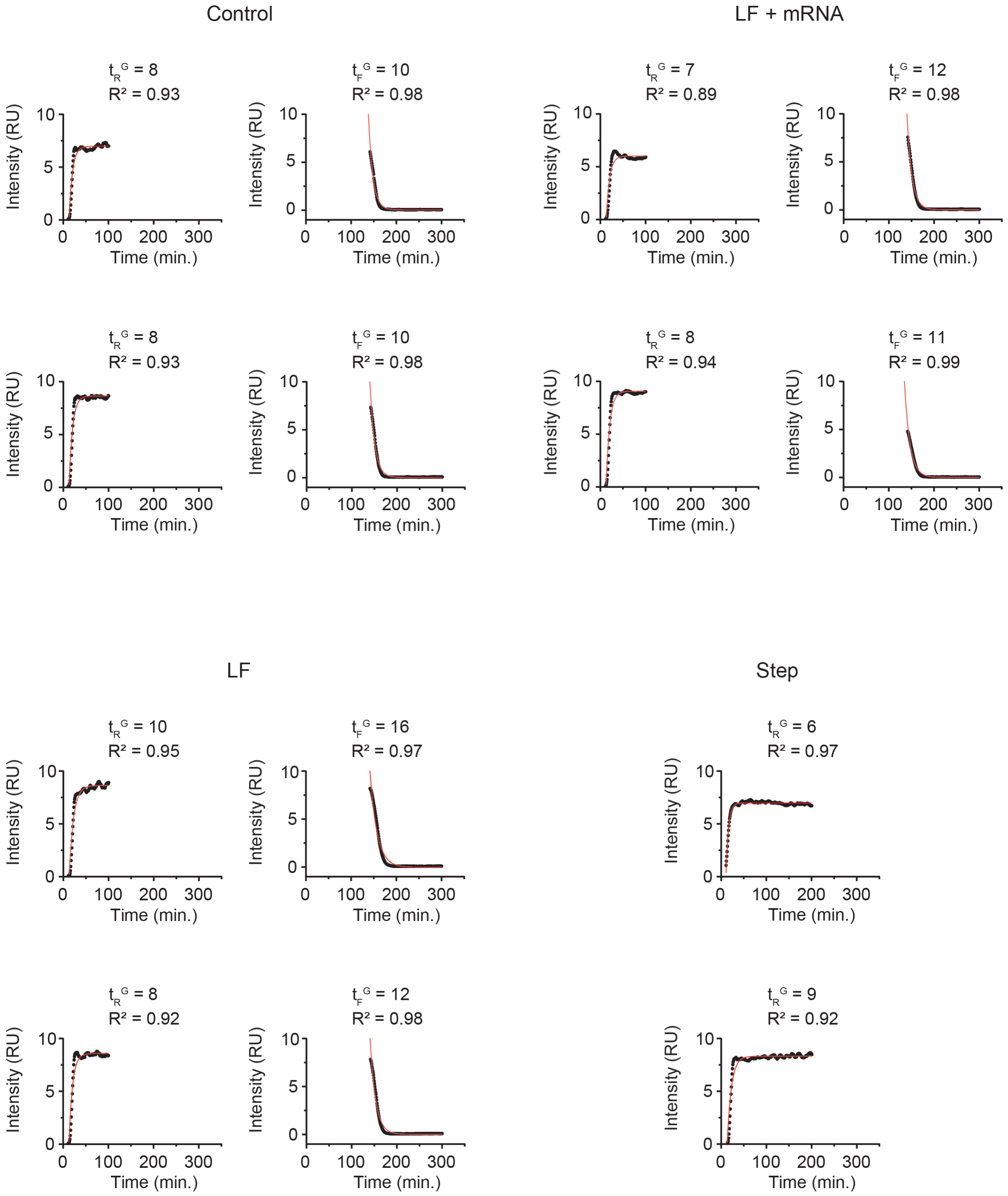

Supplementary Figure 4. Leading and trailing edge analysis of glucose pulse profiles. t_R^G and t_F^G are the rise and fall times of the ^{13}C-labeled glucose. Black traces are data from biological replicate samples and red traces indicate fits.

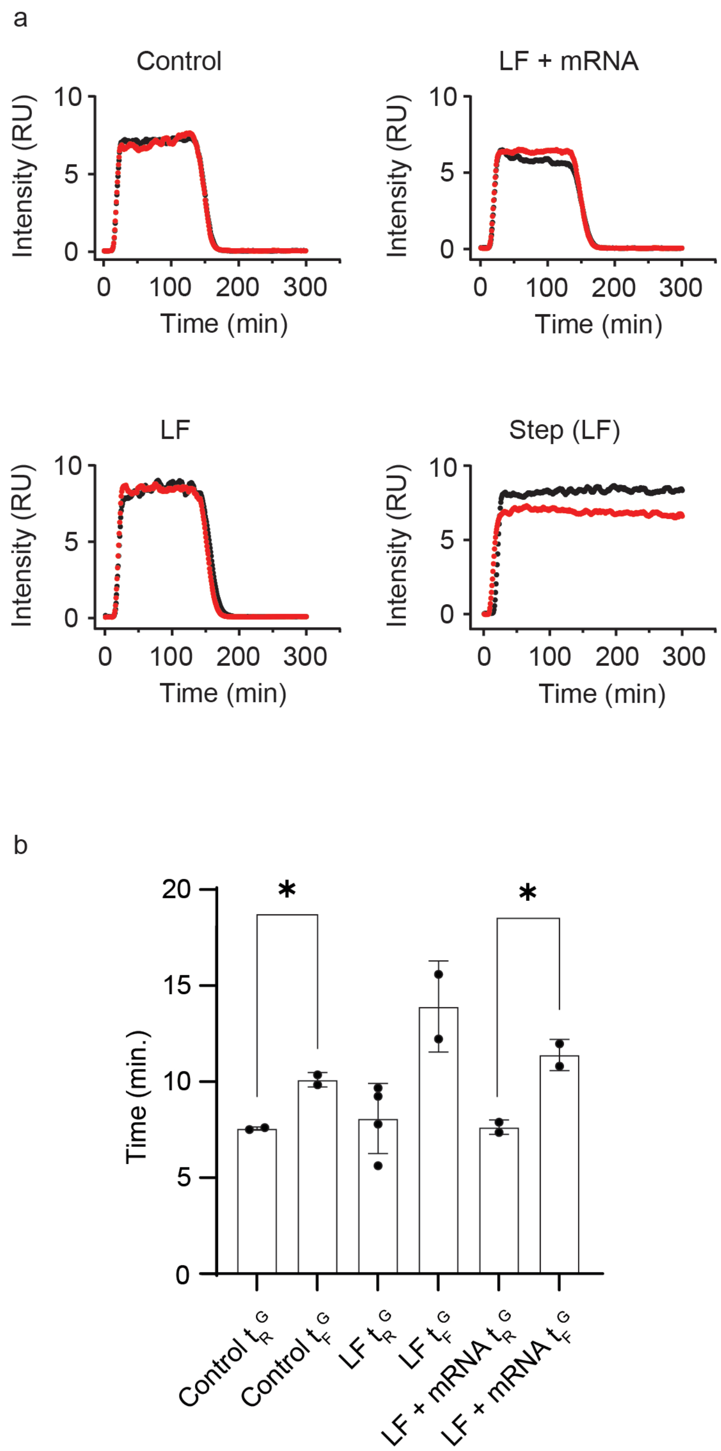

Supplementary Figure 5. Statistical analysis of glucose pulses. a). Glucose pulse profiles. Red and black traces are biological replicate samples. b). Statistical evaluation of differences between rise and fall parameters. Stars indicate significance: p-value <.05 (*), p-value <.01 (**), and p-value <.001 (***).

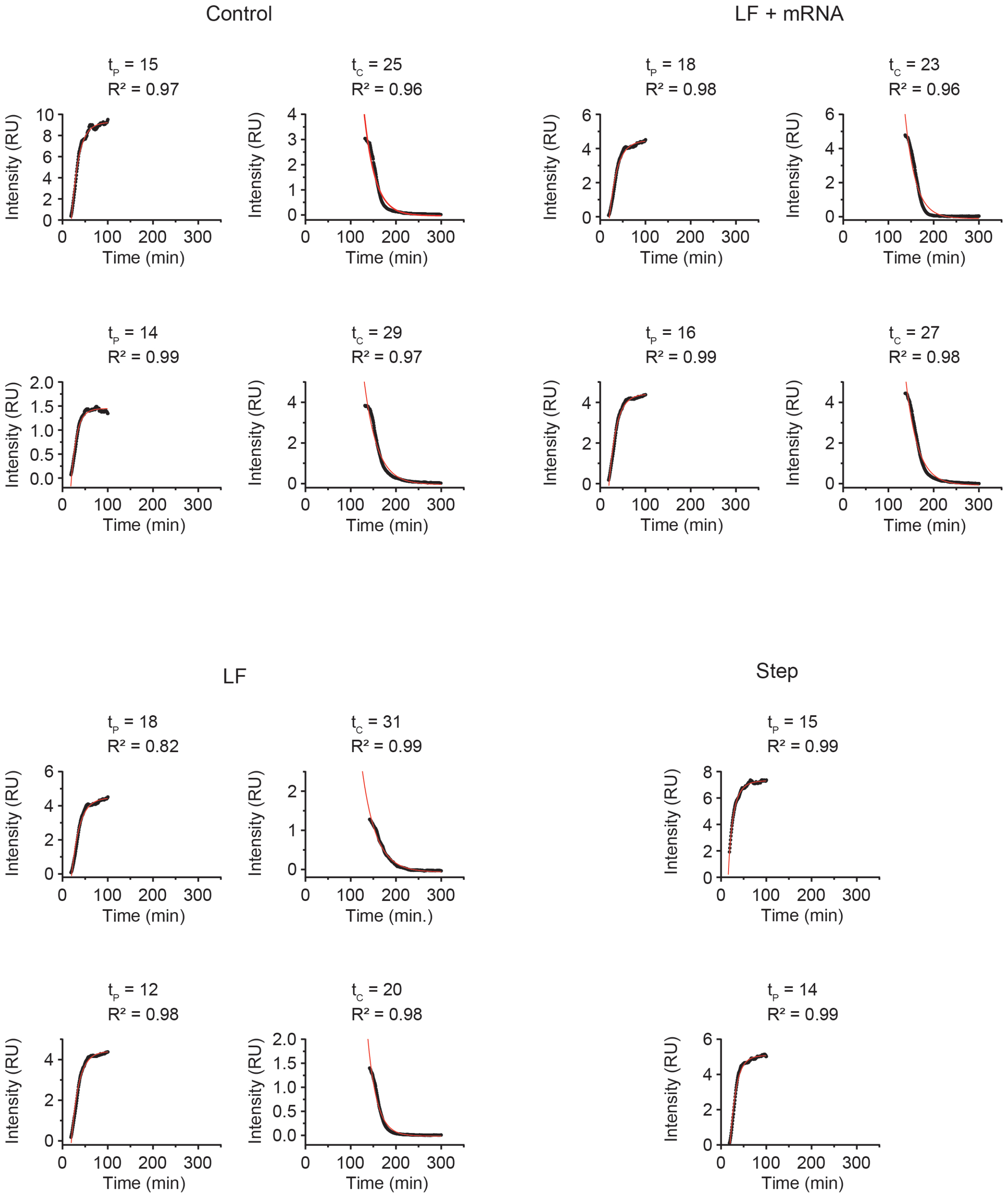

Supplementary Figure 6. Leading and trailing edge analysis of lactate profiles. Time constants for production, t_P, and clearance, t_C, and fitted curves are shown. The leading edge of the step function was obtained for cells treated with LF. Black traces are data from biological replicate samples and red traces indicate fits.

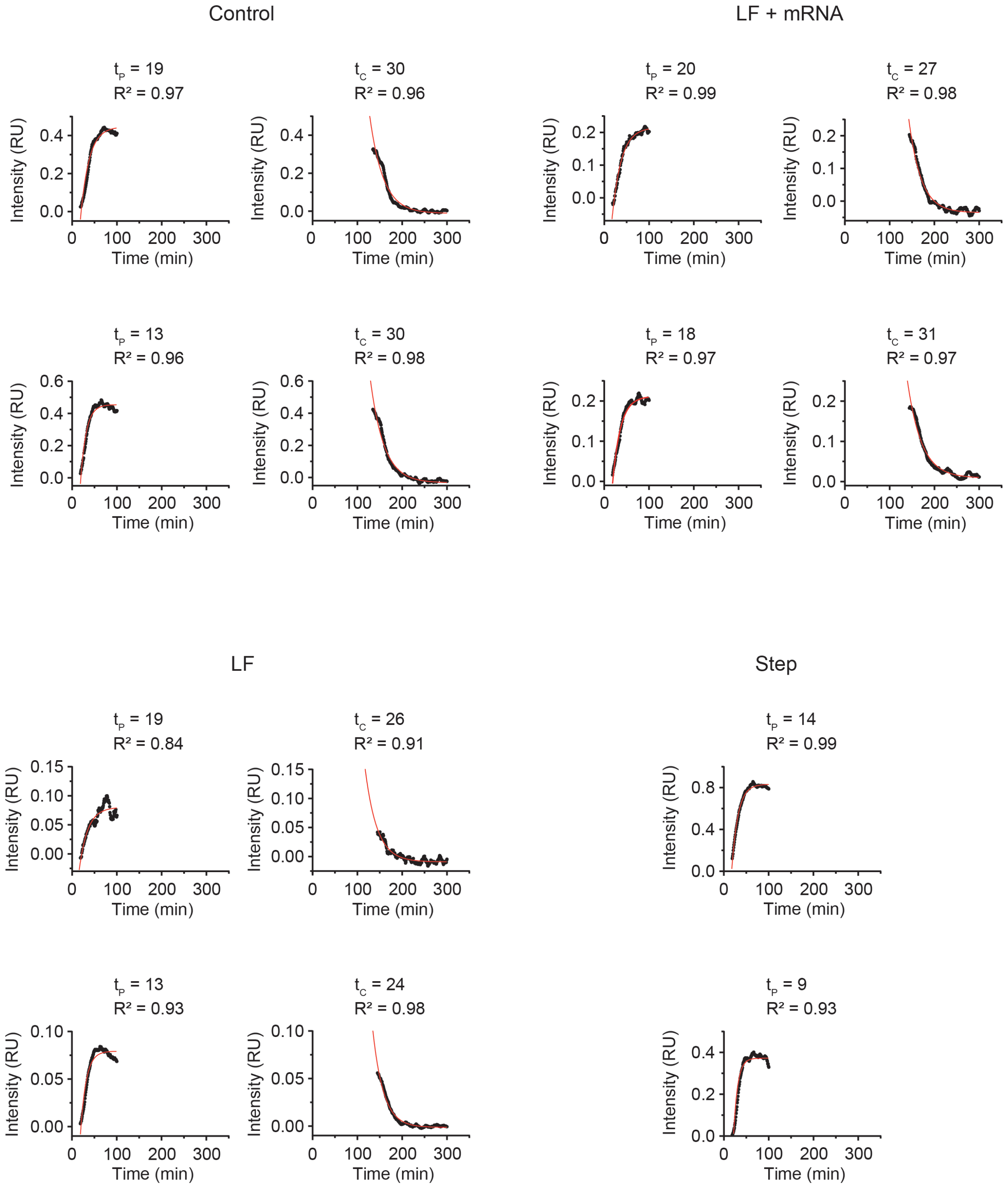

Supplementary Figure 7. Leading and trailing edge analysis of alanine profiles. Time constants for production, t_P, and clearance, t_C, and fitted curves are shown. The leading edge of the step function was obtained for cells treated with LF. Black traces are data from biological replicate samples and red traces indicate fits.

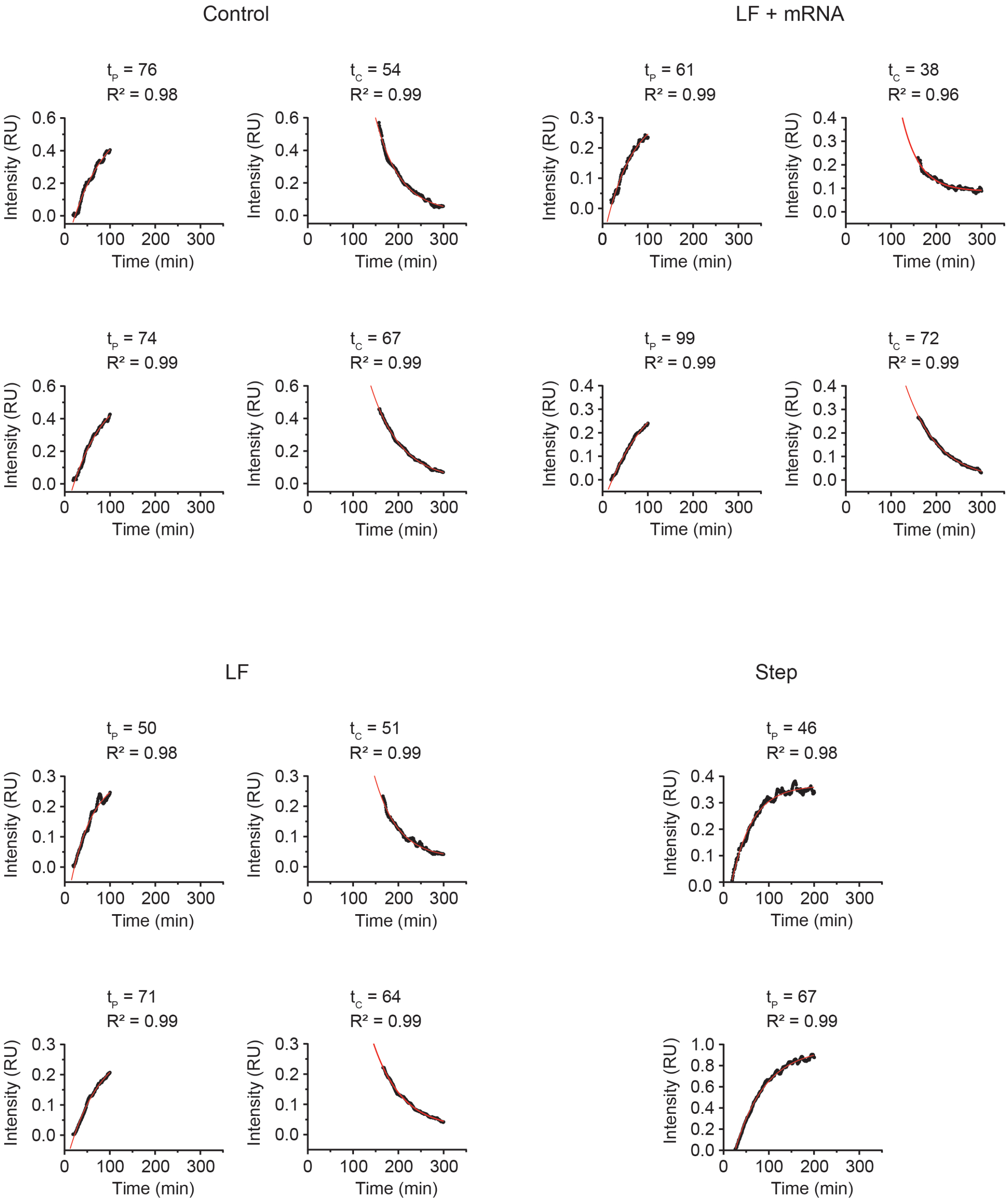

Supplementary Figure 8. Leading and trailing edge analysis of glutamate profiles. Time constants for production, t_P, and clearance, t_C, and fitted curves are shown. The leading edge of the step function was obtained for cells treated with LF. Black traces are data from biological replicate samples and red traces indicate fits.

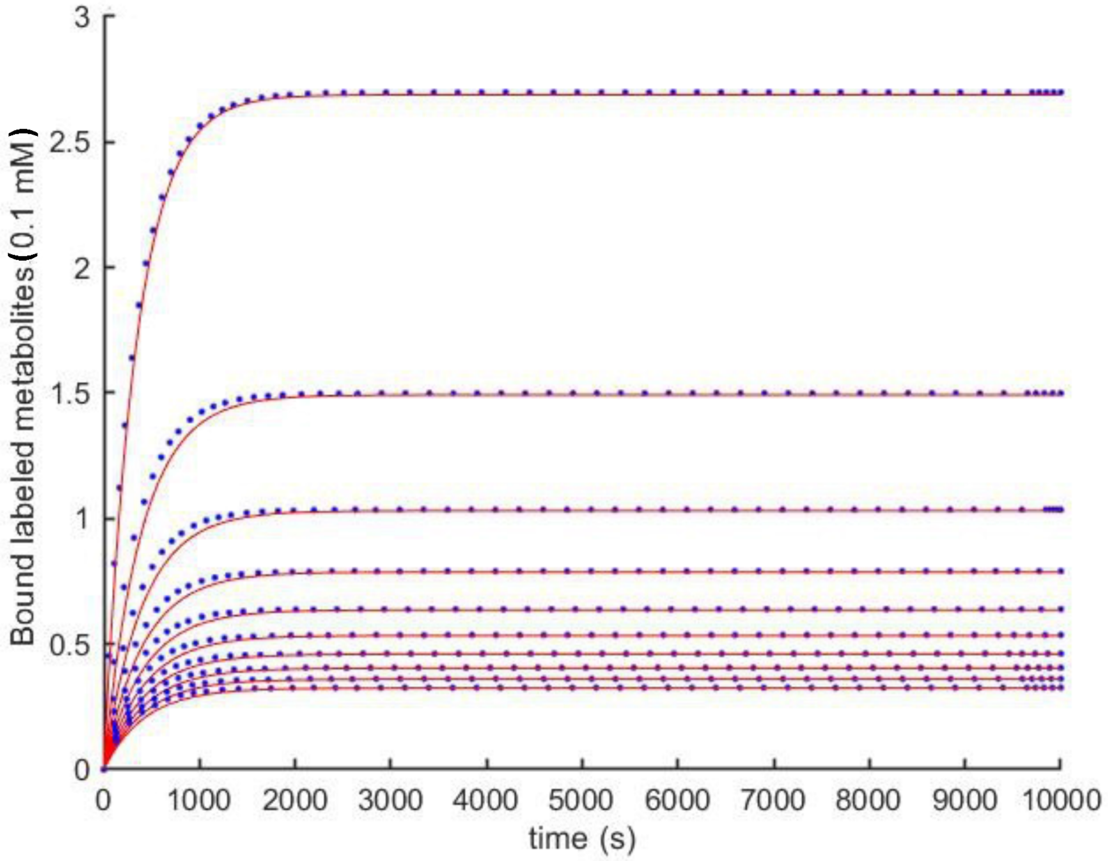

Supplementary Figure 9. Concentrations of bound labeled metabolites as a function of binding site concentration. The concentration of bound metabolites was determined by solving the equations for competitive binding of labeled metabolites at a total concentration of 1 mM in the presence of unlabeled metabolites at a concentration of 1 mM, equations 14 and 15, and varying the concentrations of binding sites from 1 mM (top curve) to 0.1 mM (bottom curve) in 0.1 mM steps. Binding rate constants were k_{on} =1 $M^{-1}s^{-1}$, k_{off} = 10^{-4} s^{-1}. The data were fit with an exponential function. The R-square measure of the goodness of fit was greater than 0.99 for all fits. The calculations were performed by using Matlab (Mathworks).

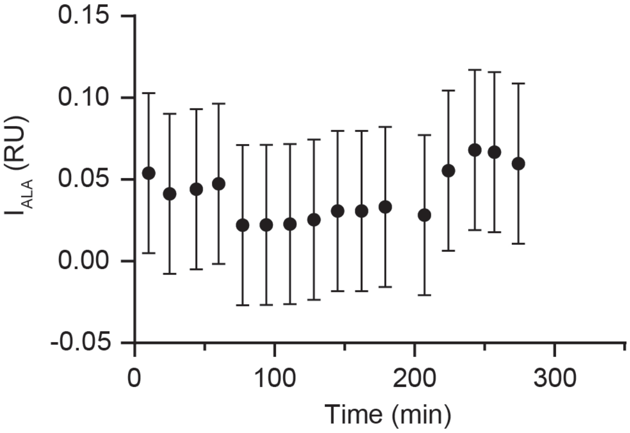

Supplementary Figure 10. Alanine excreted from cells treated with LF. The intensity of the ^{13}C-edited proton methyl peak of alanine in the flow through was monitored during the step function experiment. The alanine peak intensity was normalized to the intensity of the HEPES peak. Error bars were determined by the background noise level of the experiment.

Supplementary Tables

Supplementary Table 1. Raw data for Figure 1a.

Transfection Reagent	Average luminescence (RLU)
Fug6	11 ±0
FugHD	12 ±0
VF	12 ±1
LF	163 ±30
PEI	11 ±1
Control	11 ±1

Fug6 is FuGENE 6, FugHD is FuGENE HD, VF is ViaFect, LF is Lipofectamine 2000, and PEI is linear polyethylenimine.

Supplementary Table 2. Raw data for Figure 1b.

Time (h)	LF delivery (Log RLU)	VECT Delivery (Log RLU)
1	4.8	3.2
2	5.4	1.9
4	6.0	1.5
8	6.5	1.4
13	6.7	
14		1.3
26		1.2
28	6.5	

Supplementary Table 3. Raw data for Figure 1c.

		Luminescence (x10^6 RLU)	
Post-transfection time (h)	+ mRNA	-mRNA after 8 h	-mRNA after 12 h
0.1	0.0		
8	3.2	2.9	
9		3.5	
10		3.6	
12	4.4	4.5	3.4
13			3.8
14			3.7
16	4.9	5.3	4.2
20	4.4	4.6	4.8
37	3.2	3.4	2.9
51			0.0

Supplementary Table 4. Characteristic times for the glucose pulses.

	t_R^G (min)					t_F^G (min)		
	Control	LF	LF + mRNA	Step		Control	LF	LF + mRNA
	7.5	9.7	7.4	5.6		9.8	15.6	12.0
	7.6	7.8	7.9	9.2		10.4	12.2	10.8
		5.6						
		9.2						
avg	7.6 ±0.1	8.1 ±0.9	7.6 ±0.3	7.4 ±1.8	avg	10.1 ±0.3	13.9 ±2.6	11.4 ±0.6
t_{ch} (min)	8.7 ±0.1	9.7 ±0.9	9.1 ±0.0		K (min^{-1})	0.02 ±0.0	0.03 ±0.0	0.02 ±0.0

The estimate of t_R^G for LF included two values from the step function, for a total of four measurements. All other parameter values were estimated by using two measurements. The characteristic time of the glucose flux, t_{ch}, is equal to $2/(1/t_R^G + 1/t_F^G)$. The characteristic binding parameter that describes the rate for incorporation of the labeled glucose into and release from the bound fraction, K, is equal to $(1/t_R^G - 1/t_F^G)/2$.

Errors in t_{ch} were calculated as $\sqrt{([(t_1^{-4} \times \Delta t_1^2)/(t_1^{-1} + t_2^{-1})^4] + [(t_2^{-4} \times \Delta t_2^2)/(t_1^{-1} + t_2^{-1})^4])}$ and K error was calculated as $\sqrt{((\Delta t_1/t_1^2)^2+(\Delta t_2/t_2^2)^2)}$, where t_1 is the average t_R^G and t_2 is the average t_C^G, Δt_1 and Δt_2 refer to the difference between individual data points and the average value for the rise and fall, respectively.

Supplementary Table 5. t_P and t_C values resolved from metabolite profiles.

		t_P (min)				t_C (min)		
		Control	LF	LF + mRNA		Control	LF	LF + mRNA
Lac		14.57	17.56	17.58		24.50	30.75	23.13
		14.40	12.33	16.43		28.79	20.04	26.52
			15.50					
			13.52					
	avg	14.49 ±0.09	14.60 ±1.13	17.01 ±0.58	avg	26.65 ±2.15	25.40 ±5.36	24.83 ±1.70
Ala		13.17	18.90	20.42		30.09	26.47	27.28
		12.55	12.55	17.72		30.30	23.73	31.24
			13.15					
			9.15					
	avg	12.86 ±0.31	13.44 ±2.02	19.07 ±1.35	avg	30.20 ±0.11	25.10 ±1.37	29.26 ±1.98
Glu		76.47	50.24	61.05		54.07	51.46	38.40
		73.99	71.19	98.52		67.01	63.95	71.82
			45.55					
			66.57					
	avg	75.23 ±1.24	58.39 ±6.21	79.79 ±18.74	avg	60.54 ±6.47	57.71 ±6.25	55.11 ±6.71

Error was calculated as SEM.

Supplementary Table 6. Slopes of alanine plateau profiles.

	WT	LF	LF + mRNA
	-0.002	-0.006	-0.001
	-0.002	-0.004	-0.001
avg	-0.002<br>±0.000	-0.005<br>±0.001	-0.001<br>±0.000

Pulse windows were selected from minute 75 and 150 to account for the flattest portion of the curve and normalized to the largest value. Slopes were calculated using Prism.

Supplementary Table 7. Ratios of A_{Ro}/A_P and A_{Fo}/A_C.

	A_{Ro}/A_P			A_{Fo}/A_C		
	Control	LF	LF + mRNA	Control	LF	LF + mRNA
Lac	$1.19x10^{-13}$	$1.74x10^{-5}$	$3.16x10^{-14}$	$3.85x10^{-32}$	$3.52x10^{-32}$	$4.12x10^{-32}$
	$5.39x10^{-14}$	$4.93x10^{-32}$	$1.87x10^{-15}$	$3.21x10^{-32}$	$4.36x10^{-32}$	$3.68x10^{-32}$
Ala	$2.02x10^{-15}$	$6.96x10^{-5}$	$2.26x10^{-28}$	negligible	$4.82x10^{-32}$	$3.92x10^{-32}$
	$6.43x10^{-13}$	$6.06x10^{-12}$	$2.69x10^{-32}$	negligible	$3.62x10^{-32}$	negligible
Glu	$4.48x10^{-32}$	$4.21x10^{-32}$	$2.91x10^{-9}$	negligible	$3.91x10^{-32}$	negligible
	$3.52x10^{-32}$	$3.47x10^{-32}$	$4.27x10^{-32}$	negligible	negligible	negligible

Values are shown for fits to each biological replicate sample. The ratios that could not be defined by the fitting program are indicated as negligible.
